# Enzymatic activity necessary to restore the lethality due to *Escherichia coli* RNase E deficiency is distributed among bacteria lacking RNase E homologues

**DOI:** 10.1371/journal.pone.0177915

**Published:** 2017-05-18

**Authors:** Masaru Tamura, Daisuke Kageyama, Naoko Honda, Hirofumi Fujimoto, Atsushi Kato

**Affiliations:** 1 Department of Quality Assurance and Radiological Protection, National Institute of Infectious Diseases, Toyama, Shinjuku-ku, Tokyo, Japan; 2 Institute of Agrobiological Sciences, National Agriculture and Food Research Organization, Owashi, Tsukuba, Ibaraki, Japan; Keio University, JAPAN

## Abstract

*Escherichia coli* RNase E (Eco-RNase E), encoded by *rne* (Eco-*rne*), is considered the global RNA decay initiator. Although Eco-RNase E is an essential gene product in *E*. *coli*, some bacterial species, such as *Bacillus subtilis*, do not possess Eco-RNase E sequence homologues. *B*. *subtilis* instead possesses RNase J1/J2 (Bsu-RNase J1/J2) and RNase Y (Bsu-RNase Y) to execute RNA decay. Here we found that *E*. *coli* lacking the Eco-*rne* gene (Δ*rne E*. *coli*) was viable conditional on M9 minimal media by introducing Bsu-RNase J1/J2 or Bsu-RNase Y. We also cloned an extremely short Eco-RNase E homologue (Wpi-RNase E) and a canonical sized Bsu-RNase J1/J2 homologue (Wpi-RNase J) from *Wolbachia pipientis*, an α-proteobacterial endosymbiont of arthropods. We found that Wpi-RNase J restored the colony-forming ability (CFA) of Δ*rne E*. *coli*, whereas Wpi-RNase E did not. Unexpectedly, Wpi-RNase E restored defective CFA due to lack of Eco-RNase G, a paralogue of Eco-RNase E. Our results indicate that bacterial species that lack Eco-RNase E homologues or bacterial species that possess Eco-RNase E homologues which lack Eco-RNase E-like activities have a modest Eco-RNase E-like function using RNase J and/or RNase Y. These results suggest that Eco-RNase E-like activities might distribute among a wide array of bacteria and that functions of RNases may have changed dynamically during evolutionary divergence of bacterial lineages.

## Introduction

Studies of RNA decay began with the discovery of unstable mRNA in *Escherichia coli* [1, 2]. RNA decay investigations over many decades have demonstrated its important role in the post-transcriptional regulation of gene expression to adapt to environmental change. *E*. *coli* RNase E (Eco-RNase E) encoded by the essential gene *rne* (Eco-*rne*) was initially discovered as an endoribonuclease that processes 9S rRNA into mature 5S rRNA [3]. A sequence homologous to RNase E is conserved in various bacterial species [4, 5] (see [6] for review). RNase E targets various RNA species for cleavage, having a multifaceted role in the cleavage of structural types of RNA (5S rRNA [3], 16S rRNA [7, 8] and tRNAs [9–11]), the degradation of various types of mRNA [12–15], the regulation of plasmid DNA replication [16], and the processing of small catalytic RNAs [16, 17]. Recent studies have shown that the cleavage of certain mRNAs by RNase E is regulated by small RNAs [18–20]. The initial cleavage of RNA by RNase E is often followed by further digestion by other ribonucleases [21] (see [22] for review), thereby suggesting that RNase E is the global RNA decay initiator.

*E*. *coli* possesses RNase G (Eco-RNase G, also known as CafA), an RNase E paralogue [7, 8] encoded by the non-essential gene *rng* (Eco-*rng*), which is homologous to the N-terminal catalytic region of RNase E [23]. Defective growth owing to the mutation of Eco-*rne* (either temperature sensitive or deletion) can be restored by the overproduction of RNase G or its derivatives [13, 24–27], although RNase G-complemented Δ*rne E*. *coli* grows more slowly than the parental *rne*^+^ *E*. *coli* and continues to form filaments, which is a characteristic of RNase E-mutated *E*. *coli* [25, 28]. The hindered decay of various mRNAs by RNase E deficiency is also restored by the overproduction of RNase G [12, 13]. Owing to the sequence and functional similarity between RNase E and RNase G, homologues identified in other organisms are usually designated “RNase E/G family protein” in databases.

Eco-RNase E comprises 1,061 amino acid (aa) residues and forms tetrameric (two dimers) structures, which interact with several protein partners that assemble an “RNA degradosome” complex on cell membranes [29–33] (see [22] for review). The N-terminal half of RNase E (N-RNase E) includes the ribonucleolytic activity [23] and the C-terminal half includes a scaffold region responsible for binding to RNA degradosome components (see [6] for review). The discovery of the RNA degradosome in *E*. *coli* led to extensive investigation of the RNA processing enzyme complex in various organisms, including humans, plants, and archaea [34–36] (see [6] for review). Some bacterial species, such as *Bacillus subtilis*, do not possess an RNase E homologue [37], but they possess an RNase Y-based enzyme complex or RNase J with which to process and degrade RNA [38]. In *B*. *subtilis*, these enzymes are necessary for the normal growth of bacterial cells with the usual morphology, but the deletion of these enzymes is not strictly lethal [39]. Interestingly, some bacterial species, such as α-proteobacteria, possess homologues of both Eco-RNase E and *B*. *subtilis* RNase J1/J2 (Bsu-RNase J1/J2) [4, 40] (see [6] for review). Some physiological functions of α-proteobacterial RNase E and RNase J have been reported recently [41–45]. Understanding how these bacterial cells distinguish the functions of these ribonucleases is particularly interesting for RNA decay research [46].

These observations question whether Eco-RNase E is essential because the function of RNA decay is presumably an important biological feature of every living organism. For example, *B*. *subtilis* RNase III is uniquely essential owing to the effect of toxin/anti-toxin genes derived from prophage but its essentiality is not common in other bacterial species [47]. Features of Eco-RNase E-like RNA cleavage by Bsu-RNase J1 [40, 48] and RNase Y [49, 50], and the domain organization and the possible interaction partners of the *B*. *subtilis* RNase Y (Bsu-RNase Y)-based degradosome [38, 51] suggest that they have similar roles to Eco-RNase E in RNA decay. However, no experimental evidence has demonstrated the phenotypic restoration of lethality due to Eco-RNase E deficiency owing to an essential requirement for these ribonucleases. Thus, it is unclear how the essentiality for RNase E is established in *E*. *coli* and the common functions of RNase J or RNase Y as functional orthologues of RNase E in other organisms. Recently, we reported that part of the essentiality for Eco-RNase E is nutrient-dependent and that different genetic factors are necessary to support the growth of *E*. *coli* on rich medium (LB) and minimal medium (M9) [28, 52]. These results led us to investigate the capacity for *E*. *coli* Δ*rne* complementation by RNase J and RNase Y on minimal media with various carbon sources to understand the similarities between RNase E and RNase J or RNase Y.

In addition to *B*. *subtilis*, which lacks Eco-RNase E sequence homologues in its genome [37], we used the maternally transmitted endosymbiont *Wolbachia pipientis*, an α-proteobacteria member with an extremely short Eco-RNase E homologue, to perform *E*. *coli* Δ*rne* complementation experiments. Alpha-proteobacteria typically possess Eco-RNase E and Bsu-RNase J1/J2 homologues in their genomes [6], and expression of these genes in *W*. *pipientis* has been confirmed by RNA-seq [53], thereby suggesting that these enzymes are physiologically functional in *W*. *pipientis* cells. *W*. *pipientis* has one of the shortest RNase E/G family proteins among α-proteobacteria with a length of approximately 600 aa, even lacking the typical GWW motif for the common PNPase binding motif, which is generally conserved in the C-terminus among α-proteobacteria [54] (also see S1 Table). These observations led us to investigate the functional relationship between *W*. *pipientis* RNase E/G (Wpi-RNase E/G) and RNase J (Wpi-RNase J) homologues with Eco-RNase E.

In this study, we showed that both RNase J and RNase Y have a common enzymatic activity that phenotypically restored the lethality due to Eco-RNase E deficiency allowing Δ*rne E*. *coli* to grow (form colonies), thereby suggesting that the distribution of Eco-RNase E-like ribonucleolytic activity occurs in a wider range of bacterial species that harbor RNase J and/or RNase Y than previously considered.

## Materials and methods

### Isolation, maintenance, and preparation of *W*. *pipientis*

On May 19, 2014, a female adult *Eurema mandarina* (Insecta; Lepidoptera; Pieridae), formerly known as *Eurema hecabe mandarina* or *E*. *hecabe* yellow-type, was collected on the roadside of a prefectural road on Tanegashima Island, Kagoshima, Japan, where no specific permissions are required for sampling non-endangered insect species. This female was diagnosed by PCR as singly-infected with a cytoplasmic-incompatibility (CI)-inducing *W*. *pipientis* strain, *w*CI [55, 56]. This particular *W*. *pipientis w*CI strain, designated DK101, was transinfected into a silkworm *Bombyx mori* cell line, BmN4 (BmN) [57], and maintained as previously described [58], since *W*. *pipientis* cannot be cultivated in artificial media. *W*. *pipientis* genomic DNA was extracted as a mixture with *B*. *mori* chromosomal DNA and used as a DNA template for PCR, as previously described [58].

### Nucleotide sequence determination for *W*. *pipientis rne* and *rnj*

The primers used in this study are listed in Table 1. The oligonucleotide primers 5'-UTR-Wpi-rne and 3'-UTR-Wpi-rne were designed based on conserved regions outside the *W*. *pipientis rne* (Wpi-*rne*) open reading frame (ORF) based on eight *W*. *pipientis* strains, i.e., *w*Mel, *w*Ri, *w*Ha, *w*No, *w*Pip, *w*Bm, *w*Oo, and *w*Cle, the sequences for which were obtained from the KEGG Genes Database (http://www.genome.jp/kegg/). PCR amplification was performed using the total DNA extracted from DK101-infected BmN4 cells. The nucleotide sequence of the complete Wpi-*rne* ORF was determined using two independent DNA isolates: (i) by directly sequencing the amplified PCR products using the primers 5'-UTR-Wpi-rne and 3'-UTR-Wpi-rne, including the 5'-untranslated region (UTR) and 3'-UTR; and (ii) by sequencing pLAC-Wpi-rne, which was constructed using the primer set 5'-NotI-Wpi-rne and 3'-SpeI-Wpi-rne, to confirm that the two sequencing results were consistent (GenBank accession number LC177346 for Wpi-*rne*). The same strategy was used to determine the nucleotide sequence of the complete *W*. *pipientis rnj* (Wpi-*rnj*) ORF using the oligonucleotide primers 5'-UTR-Wpi-rnj and 3'-UTR-Wpi-rnj (GenBank accession number LC177347 for Wpi-*rnj*). Amplification of the PCR bands for Wpi-*rne* and Wpi-*rnj* was confirmed only by DK101-infected BmN4 cells, which showed that the band was derived from DK101 (S1a and S1b Fig).

**Table 1:** Primer names and sequences used in this study.

Primer name	Primer sequence
5'-UTR-Wpi-rne	5'-AGCAAAGAAGCATTTTTTTTCGCCATA-3'
3'-UTR-Wpi-rne	5'-GCAACACAAGAATTTGTTGTTCCGAGATCTAT-3'
5'-UTR-Wpi-rnj	5'-AAAGTGATAAATTCCTTTATTTA-3'
3'-UTR-Wpi-rnj	5'-ACGGATTCCAGTATTGCATGCTG-3'
5'-NotI-rne1	5'-GGATCCGCGGCCGCTTTAAGAAGGAGATATACATATGAAAAGAATGTTAATC-3'
3'-XbaSpe-rne1	5'-GTCTAGACTAGTGAATTCACTCAACAGGTTGCGGACGCG-3'
5'-NotI-Wpi-rne	5'-GGATCCGCGGCCGCTTTAAGAAGGAGATATACATATGGTGAGTGATGGCAAAAGG-3'
3'-SpeI-Wpi-rne	5'-GTCTAGACTAGTGAATTCAGTTGTTAGAGCCCAAAAGGC-3'
5'-NotI-ppsAnRBS-Wpi-rne	5'-GGATCCGCGGCCGCTATCACAAAAGGATTGTTCGATGGTGAGTGATGGCAAAAGG-3'
5'-NotI-Wpi-rnj	5'-GGATCCGCGGCCGCTTTAAGAAGGAGATATACATATGAACATAAACAAAAATGAGTTTTT-3'
3'-SpeI-Wpi-rnj	5'-GTCTAGACTAGTGAATTCATACCTGTTCTATTTGGACTT-3'
5'-Wpi-rnj-D77K-H78A	5'-ACACATGCACATGAAAAGGCCTGTGGTGCAGTGCCT-3'
3'-Wpi-rnj-D77K-H78A	5'-AGGCACTGCACCACAGGCCTTTTCATGTGCATGTGT-3'
5'-NotI-rnjA	5'-GGATCCGCGGCCGCTTTAAGAAGGAGATATACATATGAAATTTGTAAAAAATGATCAGAC-3'
3'-SpeI-rnjA	5'-GTCTAGACTAGTGAATTCAAACCTCCATAATGATCGGCA-3'
5'-rnjA-D78K-H79A	5'-ACCCACGGGCACGAAAAGGCCATCGGCGGTATTCCA-3'
3'-rnjA-DH-left	5'-GATAAAAAGCCCTTTAATTTTAT-3'
5'-NotI-rnjB	5'-GGATCCGCGGCCGCTTTAAGAAGGAGATATACATATGAAAAAGAAAAATACAGAAAACGT-3'
3'-SpeI-rnjB	5'-GTCTAGACTAGTGAATTCATACTTCCATAATAATTGGGA-3'
5'-NotI-ymdA	5'-GGATCCGCGGCCGCTTTAAGAAGGAGATATACATATGACCCCAATTATGATG-3'
3'-SpeI-ymdA	5'-GTCTAGACTAGTGAATTCATTTTGCATACTCTACGGCTC-3'
5'-ymdA-H368A-D369A-3	5'-GGGTCTTCTTGCCGCCATCGGGAAAGCAATTGACC-3'
3'-ymdA-HD-left-3	5'-GCACGTTTAGCAAGCTTTGCGTC-3'
5'-UTR-Msm-rnj	5'-AGATCCCGGCCACCACAGAAGAA-3'
3'-UTR-Msm-rnj	5'-GTATGTCGCGTTGGAGGTGCTCA-3'
5'-NotI-Msm-rnj	5'-GGATCCGCGGCCGCTTTAAGAAGGAGATATACATATGAGCGCCGAACTCGCG-3'
3'-SpeI-Msm-rnj	5'-GTCTAGACTAGTGAATTCAGATCTCTATGACGGTCGGGA-3'
5'-Msm-rnj-D85K-H86A	5'-ACCCACGCGCACGAGAAGGCCATCGGCGCGATCCCG-3'
3'-Msm-rnj-DH-left	5'-GACCACGAGCGCCTCGATCTCGT-3'

### Bacterial strains and plasmids

The bacterial strains used in this study are listed in Table 2. As the base strain, we used an *E*. *coli* strain, CM2100, where a chromosomal deletion in Eco-*rne* was complemented by a plasmid-borne Eco-*rne* gene under the control of an *araBAD* promoter (the kanamycin-resistant [Km^r^] plasmid pBAD-RNE) [28]. Eco-*rne* is essential for *E*. *coli* growth, so the addition of 0.1% L-(+)-arabinose to the CM2100 culture allowed *E*. *coli* to grow, whereas RNase E was depleted from the cells in the absence of L-(+)-arabinose, which led to growth cessation in both liquid and solid media (so-called *E*. *coli* Δ*rne* lethality). MT567 and MT570 were obtained by deleting the ORF of Eco-*rng* by introducing a Δ*rng*::Km PCR fragment into MT498 and MT504, respectively, followed by eliminating the Km resistance marker using pCP20, as previously described [59], and by transformation with pBAD-RNE after eliminating pCP20. MT875 was obtained by deleting the ORF of Eco-*rng* by introducing a Δ*rng*::Km PCR fragment into MG1655 harboring pKD46, followed by eliminating pKD46 plasmid by incubating at 37°C. MT912 and MT949 were constructed via the transformation of DH5α by pLAC-Wpi-rne and pLAC-Wpi-rnj, respectively. MT928, MT956, MT983, MT1070, MT1072, MT1125, MT1200, MT1266, MT1282, MT1288, and MT1315 were constructed via the transformation of CM2100 by pLAC-Wpi-rne, pLAC-Wpi-rnj, pLAC-Wpi-rnj-DHmut, pLAC-rnjA, pLAC-ymdA, pLAC-rnjB, pLAC-Msm-rnj, pLAC-Msm-rnj-DHmut, pnatRNE, pLAC-rnjA-DHmut, and pLAC-ymdA-HDmut, respectively. MT1094, MT1254, and MT1278 were obtained via the transformation of MT956, MT1072, and MT1070 with pSC101, respectively, and by replacing pBAD-RNE with a few passages on M9 minimal media plates containing glycerol as the sole carbon source (M9-glycerol) in the absence of Km. MT1113, MT1136, MT1137, MT1140, and MT1167 were obtained via the transformation of MT570 with pLAC-GFPuv, pLAC-Wpi-rnj, pLAC-rnjA, pLAC-ymdA, and pRNG2SΔH, respectively, and by replacing pNRNE4(Sm) in the presence of ampicillin (Ap) without streptomycin (Sm). MT1158 was obtained via the transformation of MT567 with pLAC-rnjA and by replacing pNRNE4(Sm) in the presence of Ap without Sm. MT1163 and MT1169 were constructed by culturing MT567 and MT570 in the presence of 0.1% L-(+)-arabinose without Sm to dilute out pNRNE4(Sm). MT1173, MT1176, MT1177, MT1479, MT1481, MT1483, MT1485, and MT1487 were constructed via the transformation of MT1163 by pLAC-Wpi-rnj, pLAC-rnjB, pLAC-ymdA, pLAC-GFPuv, pnatRNE, pRNG2SΔH, pLAC-rnjA, and pLAC-Wpi-rne, respectively. MT1285 was obtained via the transformation of MT1169 with pLAC-ppsAnRBS-Wpi-rne.

**Table 2:** Bacterial strains and plasmids.

Strain or plasmid	Description	Reference or source
Strains		
*Escherichia coli*		
DH5α	*deoR supE*44 *hsdR*17(r_K_-, m_K_+) *phoA recA*1 *endA*1 *gyrA*96 *thi-*1 *relA*1 Δ*(lacZYA-argF)*U169 φ80d*lacZΔ*M15	Laboratory Collection
MG1655	*ilvG rfb-*50 *rph-*1 *fnr-*267 *eut*	*E*. *coli* Genetic Stock Center (CGSC6300)
CM2100	Same as MG1655 but *rne*::cat [pBAD-RNE]	[28]
MT244	Same as CM2100 but *ydfV*::Tn10	[60]
MT498	Same as MG1655 but *rne*::cat [pNRNE4(Sm)][pKD119]	[60]
MT504	Same as MG1655 but *rne*::cat *ydfV*::Tn10 [pNRNE4(Sm)][pKD46]	[60]
MT567	Same as MG1655 but *rne*::cat Δ*rng*::FRT [pNRNE4(Sm)][pBAD-RNE]	This study
MT570	Same as MG1655 but *rne*::cat *ydfV*::Tn10 Δ*rng*::FRT [pNRNE4(Sm)][pBAD-RNE]	This study
MT658	Same as MT244 but [pLAC-GFPuv]	[60]
MT696	Same as CM2100 but [pLAC-GFPuv]	[60]
MT875	Same as MG1655 but Δ*rng*::Km	This study
MT912	Same as DH5α but [pLAC-Wpi-rne]	This study
MT928	Same as CM2100 but [pLAC-Wpi-rne]	This study
MT949	Same as DH5α but [pLAC-Wpi-rnj]	This study
MT956	Same as CM2100 but [pLAC-Wpi-rnj]	This study
MT983	Same as CM2100 but [pLAC-Wpi-rnj-DHmut]	This study
MT1070	Same as CM2100 but [pLAC-rnjA]	This study
MT1072	Same as CM2100 but [pLAC-ymdA]	This study
MT1094	Same as MG1655 but *rne*::cat [pSC101][pLAC-Wpi-rnj]	This study
MT1113	Same as MT658 but Δ*rng*::FRT	This study
MT1125	Same as CM2100 but [pLAC-rnjB]	This study
MT1136	Same as MT244 but Δ*rng*::FRT [pLAC-Wpi-rnj]	This study
MT1137	Same as MT244 but Δ*rng*::FRT [pLAC-rnjA]	This study
MT1140	Same as MT244 but Δ*rng*::FRT [pLAC-ymdA]	This study
MT1158	Same as CM2100 but Δ*rng*::FRT [pLAC-rnjA]	This study
MT1163	Same as CM2100 but Δ*rng*::FRT	This study
MT1167	Same as MT244 but Δ*rng*::FRT [pRNG2SΔH]	This study
MT1169	Same as MT244 but Δ*rng*::FRT	This study
MT1173	Same as MT1163 but [pLAC-Wpi-rnj]	This study
MT1176	Same as MT1163 but [pLAC-rnjB]	This study
MT1177	Same as MT1163 but [pLAC-ymdA]	This study
MT1200	Same as CM2100 but [pLAC-Msm-rnj]	This study
MT1254	Same as MG1655 but *rne*::cat [pSC101][pLAC-ymdA]	This study
MT1266	Same as CM2100 but [pLAC-Msm-rnj-DHmut]	This study
MT1278	Same as MG1655 but *rne*::cat [pSC101][pLAC-rnjA]	This study
MT1282	Same as CM2100 but [pnatRNE]	This study
MT1285	Same as MT1169 but [pLAC-ppsAnRBS-Wpi-rne]	This study
MT1288	Same as CM2100 but [pLAC-rnjA-DHmut]	This study
MT1315	Same as CM2100 but [pLAC-ymdA-HDmut]	This study
MT1479	Same as MT1163 but [pLAC-GFPuv]	This study
MT1481	Same as MT1163 but [pnatRNE]	This study
MT1483	Same as MT1163 but [pRNG2SΔH]	This study
MT1485	Same as MT1163 but [pLAC-rnjA]	This study
MT1487	Same as MT1163 but [pLAC-Wpi-rne]	This study
*Bacillus subtilis*		
168	*trpC2*	Bacillus Genetic Stock Center (BGSC1A1)
*Wolbachia pipientis*		
DK101	Strain *w*CI(63–19) extracted from *Eurema mandarina*	This study
*Mycobacterium smegmatis*		
MC^2^ 155	Ept^-^	[82]
Plasmids		
pBAD-RNE	pSC101 *ori* Km^r^, *E*. *coli rne* under P_BAD_	[13,25]
pNRNE4	P15A *ori* Ap^r^, *E*. *coli* His-tagged N-*rne* under *lac*UV5 promoter	[25]
pNRNE4(Sm)	P15A *ori* Sm^r^, *E*. *coli* His-tagged N-*rne* under *lac*UV5 promoter	[60]
pnatRNE	P15A *ori* Ap^r^, *E*. *coli rne* under *lac*UV5 promoter	This study
pRNG2SΔH	P15A *ori* Ap^r^, *E*. *coli* natural short form *rng* under *lac*UV5 promoter	[28]
pLAC-GFPuv	P15A *ori* Ap^r^, *gfpuv* under *lac*UV5 promoter	[60]
pLAC-Wpi-rne	P15A *ori* Ap^r^, *W*. *pipientis rne* under *lac*UV5 promoter	This study
pLAC-ppsAnRBS-Wpi-rne	P15A *ori* Ap^r^, *W*. *pipientis rne* with *ppsA* 5'-UTR under *lac*UV5 promoter	This study
pLAC-Wpi-rnj	P15A *ori* Ap^r^, *W*. *pipientis rnj* under *lac*UV5 promoter	This study
pLAC-Wpi-rnj-DHmut	P15A *ori* Ap^r^, *W*. *pipientis rnj* with D77K and H78A substitution under *lac*UV5 promoter	This study
pLAC-rnjA	P15A *ori* Ap^r^, *B*. *subtilis rnjA* under *lac*UV5 promoter	This study
pLAC-rnjA-DHmut	P15A *ori* Ap^r^, *B*. *subtilis rnjA* with D78K and H79A substitution under *lac*UV5 promoter	This study
pLAC-rnjB	P15A *ori* Ap^r^, *B*. *subtilis rnjB* under *lac*UV5 promoter	This study
pLAC-ymdA	P15A *ori* Ap^r^, *B*. *subtilis ymdA* under *lac*UV5 promoter	This study
pLAC-ymdA-HDmut	P15A *ori* Ap^r^, *B*. *subtilis ymdA* with H367A and D368A substitution under *lac*UV5 promoter	This study
pLAC-Msm-rnj	P15A *ori* Ap^r^, *M*. *smegmatis rnj* under *lac*UV5 promoter	This study
pLAC-Msm-rnj-DHmut	P15A *ori* Ap^r^, *M*. *smegmatis rnj* with D85K and H86A substitution under *lac*UV5 promoter	This study
pSC101	pSC101 *ori* Tc^r^	[71,72]
pKD46	*oriR101* repA101(ts) Ap^r^ *araC*^+^ P_BAD_-Red	[59]
pKD119	*oriR101* repA101(ts) Tc^r^ *araC*^+^ P_BAD_-Red	[59]
pCP20	pSC101(ts) *ori* Ap^r^ Cm^r^ cI857 P_r_-FLP	[83]

pnatRNE, which expresses a full-length Eco-RNase E, was constructed by ligating *Not*I- and *Spe*I-digested PCR products encoding the region of the full-length Eco-*rne* into pNRNE4 [25], using the same restriction enzyme sites, thereby replacing the N-RNase E coding region from pNRNE4 with the full-length Eco-RNase E coding region. The Eco-*rne* fragment was amplified by PCR using the primers 5'-NotI-rne1 and 3'-XbaSpe-rne1 with MG1655 genomic DNA as the template. pLAC-Wpi-rne expressing the natural form (i.e., no tag) of Wpi-RNase E was constructed by ligating the *Not*I- and partially *Spe*I-digested PCR products (the Wpi-*rne* ORF contains one *Spe*I site) encoding the region of Wpi-*rne* into pNRNE4 using the same restriction enzyme sites. The Wpi-*rne* fragment was amplified by PCR using the primers 5'-NotI-Wpi-rne and 3'-SpeI-Wpi-rne, with the total DNA extract from DK101-infected BmN4 as the template. pLAC-ppsAnRBS-Wpi-rne where the 5'-UTR region of Wpi-*rne* in pLAC-Wpi-rne was replaced by the 5'-UTR region of *E*. *coli ppsA* gene to decrease the expression level of Wpi-RNase E compared with pLAC-Wpi-rne, was constructed by the same method as pnatRNE, except the primers 5'-NotI-ppsAnRBS-Wpi-rne and 3'-SpeI-Wpi-rne were used with the total DNA extract from DK101-infected BmN4 as the template. pLAC-Wpi-rnj expressing Wpi-RNase J was constructed by the same method as pnatRNE, except the primers 5'-NotI-Wpi-rnj and 3'-SpeI-Wpi-rnj were used with the total DNA extract from DK101-infected BmN4 as the template. pLAC-Wpi-rnj-DHmut expressing Wpi-RNase J where aspartic acid 77 (GAT) and histidine 78 (CAC) were substituted for lysine (AAG) and alanine (GCC), respectively, was constructed by spontaneous recircularization via the transformation of *E*. *coli* competent cells (DH5α) with the PCR product amplified using the primers 5'-Wpi-rnj-D77K-H78A and 3'-Wpi-rnj-D77K-H78A, with the pLAC-Wpi-rnj plasmid as the template. pLAC-rnjA expressing Bsu-RNase J1 was constructed by the same method as pnatRNE, except the primers 5'-NotI-rnjA and 3'-SpeI-rnjA were used with *B*. *subtilis* 168 genomic DNA as the template. pLAC-rnjA-DHmut expressing Bsu-RNase J1, with aspartic acid 78 (GAC) and histidine 79 (CAC) substituted for lysine (AAG) and alanine (GCC), respectively, was constructed via the self-ligation of the T4 polynucleotide kinase-treated PCR product amplified using the primers 5'-rnjA-D78K-H79A and 3'-rnjA-DH-left with pLAC-rnjA as the template. pLAC-rnjB expressing Bsu-RNase J2 was constructed using the same method as pLAC-rnjA, except using the primers 5'-NotI-rnjB and 3'-SpeI-rnjB. pLAC-ymdA expressing Bsu-RNase Y was constructed using the same method as pLAC-rnjA, except the primers 5'-NotI-ymdA and 3'-SpeI-ymdA were used. pLAC-ymdA-HDmut expressing Bsu-RNase Y, with both histidine 368 (CAC) and aspartic acid 369 (GAC) substituted for alanine (GCC), was constructed via the self-ligation of the T4 polynucleotide kinase-treated PCR product amplified using the primers 5'-ymdA-H368A-D369A-3 and 3'-ymdA-HD-left-3 with pLAC-ymdA as the template. pLAC-Msm-rnj, which expresses *Mycoplasma smegmatis* RNase J (Msm-RNase J), was constructed using the same method as pnatRNE, except using the primers 5'-UTR-Msm-rnj and 3'-UTR-Msm-rnj to amplify the first PCR product with *M*. *smegmatis* MC^2^ 155 genomic DNA as the template, followed by PCR amplification using the primers 5'-NotI-Msm-rnj and 3'-SpeI-Msm-rnj to amplify the second PCR product with the first PCR product as the template. pLAC-Msm-rnj-DHmut expressing Msm-RNase J, with aspartic acid 85 (GAC) and histidine 86 (CAC) substituted for lysine (AAG) and alanine (GCC), respectively, was constructed via the self-ligation of the T4 polynucleotide kinase-treated PCR product amplified using the primers 5'-Msm-rnj-D85K-H86A and 3'-Msm-rnj-DH-left with pLAC-Msm-rnj as the template.

### Media and culture conditions

The media and culture conditions were essentially those described previously [28, 60]. LB medium [61] and M9 minimal medium [62] containing 0.1 mM CaCl_2_, 1 mM MgSO_4_, and appropriate carbon sources at the following concentrations were used: glycerol, 0.5%; and sodium pyruvate, 0.2%. Appropriate antibiotics were used at the following concentrations: Ap, 50 μg/ml; chloramphenicol, 10 μg/ml; Km, 20 μg/ml; tetracycline (Tc), 5 μg/ml; and Sm, 50 μg/ml. Gellan gum (Wako 073–03071) (0.6% final concentration) was used as a gelling agent in all media plates. *E*. *coli* strains with the Δ*rne* mutation complemented with pBAD-RNE were streaked from glycerol stock (containing 40% glycerol) onto plates containing 0.1% L-(+)-arabinose and incubated overnight at 37°C, before the colonies were picked for inoculation.

### Protein analyses

Cultures of DH5α, MT912 (pLAC-Wpi-rne) or MT949 (pLAC-Wpi-rnj) cells were freshly grown to an approximate optical density at 600 nm [OD_600_] of 2.0 in LB medium containing the appropriate antibiotics but without isopropyl-β-D-thiogalactopyranoside (IPTG). Cells were diluted to an OD_600_ of 0.4 in fresh LB medium containing the appropriate antibiotics and 50 μM IPTG, before further culture for 90 min at 37°C. They were then harvested and prepared for sodium dodecyl sulfate-polyacrylamide gel electrophoresis (SDS-PAGE). Lysates containing total cellular proteins (0.07 OD_600_ units per lane) were electrophoresed on 10% polyacrylamide gel and stained with Coomassie Brilliant Blue (CBB) (Quick-CBB PLUS, Wako).

### Plating experiments

Plating experiments were essentially performed as described previously [60]. *E*. *coli* strains were streaked from glycerol stock and grown overnight at 37°C on LB plates containing appropriate supplements and antibiotics. After colonies of adequate size (approximately 1 mm in diameter) had formed, the cells were freshly grown (never grown overnight) from a single colony to the mid- to late-log phase at 37°C in LB medium containing the indicated supplements and antibiotics. Cells were diluted from 10^−4^ to 10^−5^, and 100 μl of the diluted culture was spread on LB or M9 plates containing the indicated carbon sources, supplements, and antibiotics. The plates were incubated at 37°C for 6 days, as indicated, and then scanned. As previously reported [60], the Δ*rne Δrng* double mutant *E*. *coli* grows more slowly than the Δ*rne* single mutant *E*. *coli*, and thus the incubation period was extended to 14 days (as indicated) to facilitate visualization of the colonies. The incubation conditions in each experiment are described in the figure legends. In general, the absence of detectable colony formation (with 100% colony-forming efficiency (CFE)) after incubation for 6 days was defined as a colony-forming ability (CFA)-negative phenotype.

### Statistical analyses

To infer the molecular history of Eco-RNase E homologues derived from *W*. *pipientis*, the aa sequences of RNase E/G family proteins derived from 20 bacterial representatives, including *W*. *pipientis* and *E*. *coli*, were aligned using the online program Clustal Omega [63] and subjected to phylogenetic analyses. Using MEGA version 6.06 [64], the evolutionary history was inferred by the maximum likelihood method based on the JTT matrix-based model [65]. Initial trees for the heuristic search were obtained automatically by applying the neighbor-joining (NJ) and BioNJ algorithms to a matrix of pairwise distances estimated using a JTT model, before selecting the topology with the best log-likelihood value. A discrete Gamma distribution was used to model the evolutionary rate of differences among sites. The rate variation model allowed some sites to be evolutionarily invariable. All positions containing gaps and missing data were eliminated.

To obtain evolutionary insights into the length variation in RNase E among α-proteobacterial lineages, the nucleotide sequences of the16S rRNA genes from 39 α-proteobacterial lineages with known RNase E lengths as well as from *E*. *coli* were aligned using ClustalW, and then subjected to phylogenetic analyses based on the maximum likelihood method using MEGA. Similarly, the aa sequences of RNase E from the 40 lineages were aligned using Clustal Omega and subjected to phylogenetic analyses as described above.

To infer the selective forces operating on RNase E and RNase J in *W*. *pipientis* lineages, the nucleotide sequences of the two genes from 11 lineages of *W*. *pipientis* were aligned using ClustalW, and the number of nonsynonymous substitutions per nonsynonymous site (Ka) and the number of synonymous substitutions per synonymous site (Ks) [66] were estimated using DnaSP version 5 [67].

### Nucleotide sequence accession numbers

The sequences reported in this study have been deposited in the GenBank database (http://www.ncbi.nlm.nih.gov/genbank/) under accession no. LC177346 for Wpi-*rne* and no. LC177347 for Wpi-*rnj* from *W*. *pipientis* strain DK101.

## Results

### The *W*. *pipientis* genome contains two ORFs with high similarity to Eco-RNase E and Bsu-RNase J1/J2

*B*. *subtilis* RNase J1/J2 encoded by *rnjA*/*rnjB* and RNase Y encoded by *ymdA* are well characterized [38, 40, 50]. However, genetic analyses have not been reported for the Eco-RNase E homologue or Bsu-RNase J1/J2 homologue from *W*. *pipientis*, an endosymbiont α-proteobacterium [6]. Using BLAST search [68], we found that a putative single ORF in *W*. *pipientis* in the database included a segment that shared high similarity with the catalytic domain (N-terminal 529 residues) of Eco-RNase E (87% coverage with 39% shared identity), and also that a putative single ORF in *W*. *pipientis* included a segment highly similar with the full-length Bsu-RNase J1/J2 (98% coverage with 31% shared identity for J1, and 91% coverage with 31% shared identity for J2). The transcription of these genes has been confirmed by global RNA-seq analysis [53], thereby suggesting that they are functional in *W*. *pipientis* cells. No sequences with homology to Bsu-RNase Y were found in the *W*. *pipientis* genome.

To construct plasmids that expressed each of these enzymes for use in further analyses, we determined the complete nucleotide sequences of the Eco-*rne* homologue in *W*. *pipientis* (Wpi-*rne*) and the *B*. *subtilis rnjA*/*rnjB* homologue in *W*. *pipientis* (Wpi-*rnj*). The correspondence between the sequencing results for both the PCR products and the cloned ORFs of Wpi-*rne* (pLAC-Wpi-rne) or Wpi-*rnj* (pLAC-Wpi-rnj) confirmed the presence of an ORF encoding a predicted 591-aa protein (designated as Wpi-RNase E/G) with a calculated molecular weight of 67.1 kDa and an ORF encoding a predicted 544-aa protein (designated as Wpi-RNase J) with a calculated molecular weight of 60.5 kDa (S1 Fig).

### *In silico* analyses of Wpi-RNase E/G and Wpi-RNase J

**Fig 1:**
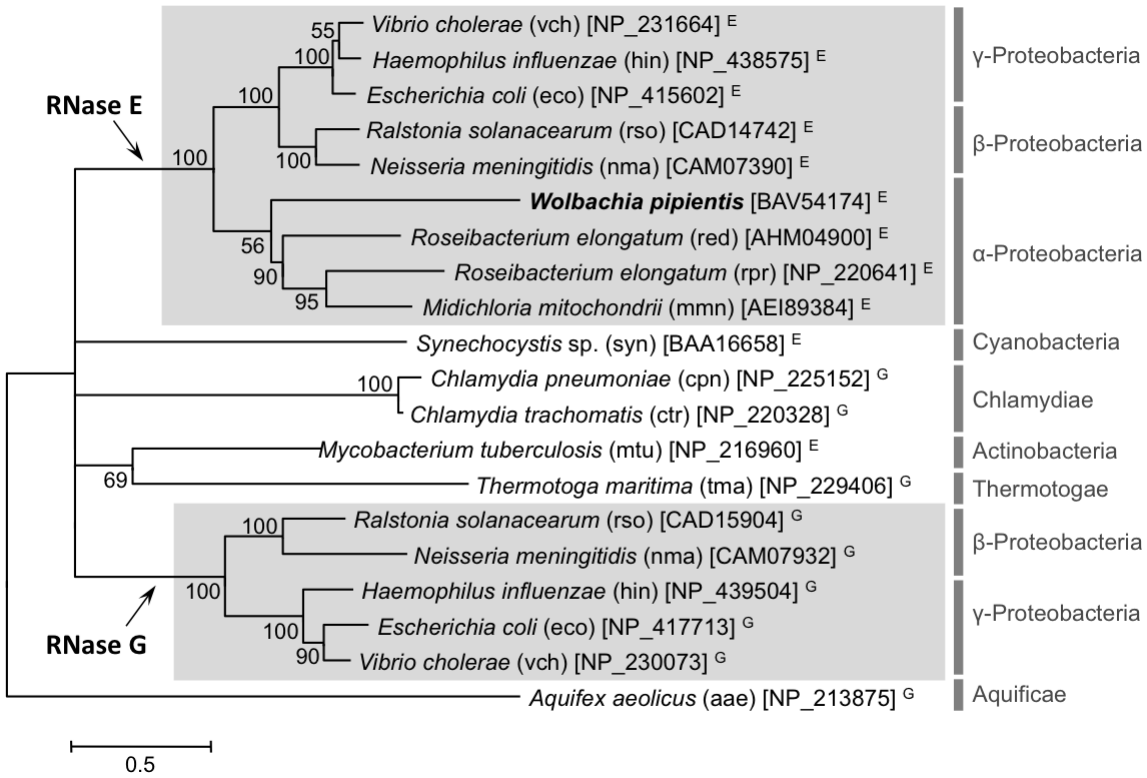
Molecular phylogenetic tree of RNase E/G family proteins derived from 15 representative bacterial lineages. The evolutionary history was inferred using the maximum likelihood method (see the Materials and methods for details). The percentage of trees in which the associated taxa clustered together (bootstrap values) is shown next to the branches. The tree is drawn to scale and the branch lengths were operationalised as number of substitutions per site. Nodes with less than 50% bootstrap support are collapsed. KEGG organism codes are given in parentheses. NCBI Protein IDs are given in square brackets. Superscripts E and G represent those that were registered on the database as RNase E and RNase G, respectively. Highly supported clades containing *E*. *coli* RNase E and RNase G are highlighted by shading.

Eco-RNase E and Eco-RNase G in the *E*. *coli* genome are homologous enzymes, which were probably derived from a single ancestral enzyme via a gene duplication event [69]. In other organisms, the gene sequences homologous to Eco-RNase E and Eco-RNase G are provisionally annotated as “RNase E/G family protein” because their enzymatic activities have not been confirmed, genetically or biochemically. Our phylogenetic analysis indicated that the RNase E/G family protein members could be separated into two clades, each of which included either Eco-RNase E or Eco-RNase G, and Wpi-RNase E/G was classified as a member of the RNase E group (Fig 1).

In agreement with the phylogenetic relationships of *W*. *pipientis* based on five housekeeping genes [70], *W*. *pipientis* strains *w*CI and *w*Pip (a close relative of *w*CI) shared the most similar RNase E sequences with each other, where both had a 3-aa deletion ca. 20 aa from the C-terminus and they possessed only two amino acid substitutions (S2 Fig). Strikingly, a 9-aa insertion (FSVRRCTHI) was observed at ca. 50 aa from the C-terminus only in *w*CI RNase E (S2 Fig). The 9-aa insertion at this position was found consistently in three isolates of *w*CI derived from three independently-collected butterflies.

To infer the selective forces that operate on Wpi-RNase E and Wpi-RNase J, we aligned and analyzed the nucleotide sequences of the two *w*CI genes and 10 other lineages of *W*. *pipientis* with published whole genome sequences. The average Ka/Ks ratios were 0.1407 for Wpi-RNase E and 0.1093 for Wpi-RNase J, thereby suggesting these are not under positive selection.

### RNase J or RNase Y is sufficient partially to reverse the effects of Eco-RNase E deficiency, thereby restoring CFA in Δ*rne E*. *coli*

**Fig 2:**
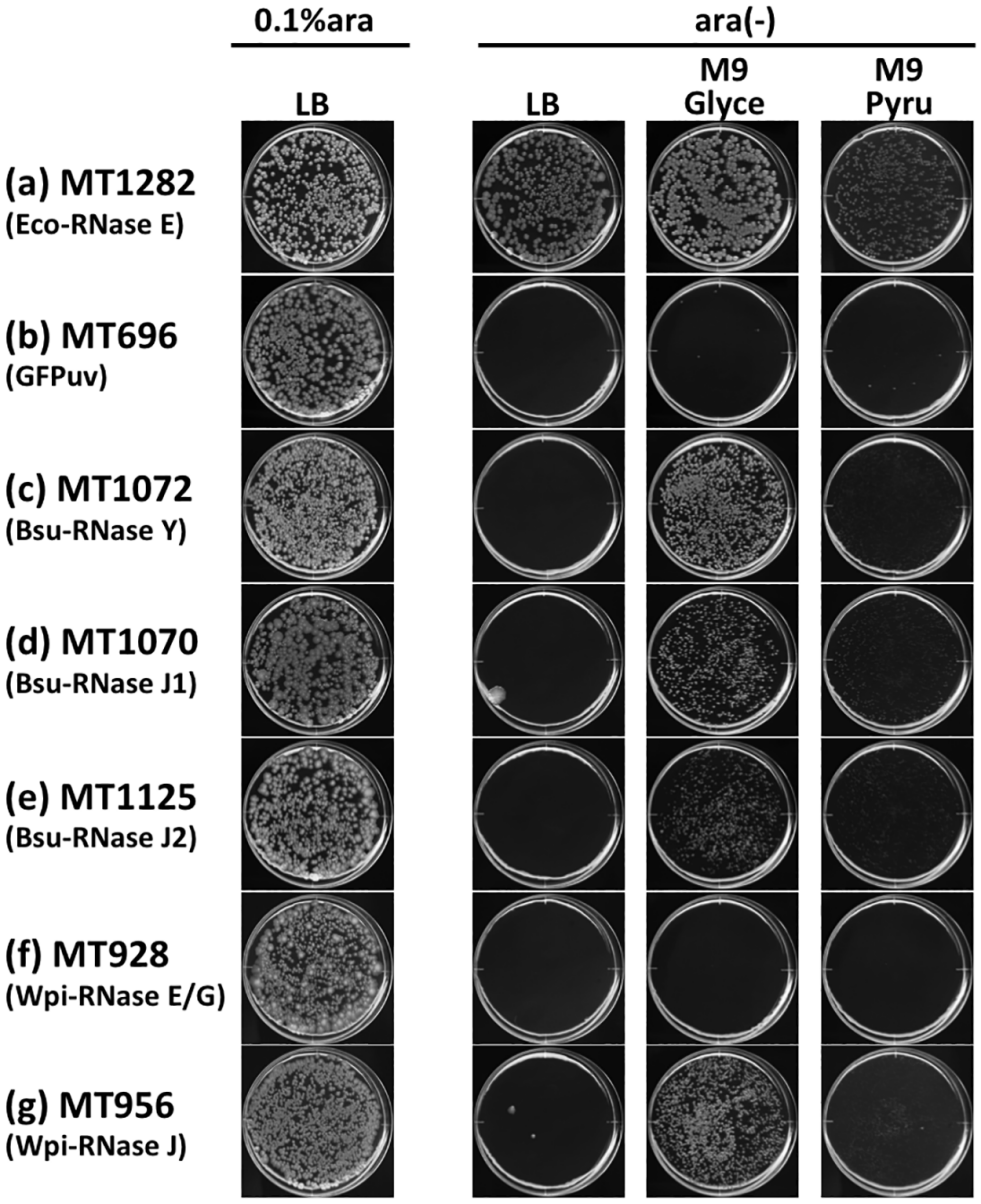
Growth of RNase E-, RNase J-, or RNase Y-complemented Δ*rne E*. *coli* strains on LB and M9 plates with various carbon sources. Cultures of MT1282 (a), MT696 (b), MT1072 (c), MT1070 (d), MT1125 (e), MT928 (f), and MT956 (g) were spread on LB or M9 gellan gum plates with various carbon sources containing (0.1% ara) or lacking [ara(-)] 0.1% L-(+)-arabinose, as indicated. Appropriate concentrations of IPTG (10 μM for MT928, MT956, and MT1125; 50 μM for MT1072; no IPTG (leaky expression) for MT696, MT1070, and MT1282) were added to the plates. Plates were scanned after incubation at 37°C for 6 days. *Glyce* glycerol, *Pyru* pyruvate.

Previously reported cleavage assays indicate that Bsu-RNase J1/J2 shares a similar RNA cleavage profile with Eco-RNase E [40, 48], while the similarity of interaction partners and degradosome assembly between Bsu-RNase Y and Eco-RNase E also suggests a functional relationship between these two enzymes [51]. However, there have been no reports of the successful growth restoration of Δ*rne E*. *coli* using these enzymes. Recently, we reported that a part of the essentiality for Eco-RNase E is nutrient-dependent and that M9 minimal media, instead of LB media, supported the CFA of various Δ*rne* revertants [28, 52]. Thus, we were interested in learning whether the similar shared profiles of RNase Y and RNase J when complementing the essentiality of Eco-RNase E depended on different carbon sources, thereby helping to visualize the functional distinction between these ribonucleases. In these experiments, an IPTG-inducible plasmid containing an ORF for Bsu-RNase Y (pLAC-ymdA), Bsu-RNase J1 (pLAC-rnjA), Bsu-RNase J2 (pLAC-rnjB), Wpi-RNase E (pLAC-Wpi-rne), or Wpi-RNase J (pLAC-Wpi-rnj) was introduced via transformation into *E*. *coli* strain CM2100 harboring the pBAD-RNE plasmid with the chromosomal *rne* deleted. The ability to complement *E*. *coli* Δ*rne* was tested by observing CFA without L-(+)-arabinose, as described previously [28, 52, 60]. Appropriate concentration of IPTG for each RNase to restore CFA of Δ*rne E*. *coli* was determined by preliminary plating experiments (S3 Fig) and the expression of GFPuv or each RNase in the complemented strain was confirmed by SDS-PAGE (S4 and S5 Figs). The CFA was restored for the full-length Eco-RNase E-complemented Δ*rne E*. *coli* on all media plates tested (Fig 2a). Colonies with approximately 1 mm in diameter could be observed within 24hr for full-length Eco-RNase E complemented Δ*rne E*. *coli* strain (MT1282). GFPuv did not restore CFA on any of the media plates tested (Fig 2b), but the restoration of CFA was observed on M9 minimal media plates when Δ*rne E*. *coli* was complemented with Bsu-RNase Y, Bsu-RNase J1, Bsu-RNase J2, or Wpi-RNase J (Fig 2c–2e and 2g), which suggests that these enzymes have an enzymatic activity similar to Eco-RNase E. Wpi-RNase E did not cause restoration on any of the media plates tested (Fig 2f). Except for the full-length Eco-RNase E, none of the complementation effects restored CFA in Δ*rne E*. *coli* on LB media plates (Fig 2).

To exclude the possibility that the restoration of CFA by Bsu-RNase Y, Bsu-RNase J1/J2, and Wpi-RNase J complementation was due to the leaky expression of Eco-RNase E from the pBAD-RNE plasmid in these cells, we eliminated the pBAD-RNE plasmid from MT1072 (Bsu-RNase Y), MT1070 (Bsu-RNase J1), MT1125 (Bsu-RNase J2), and MT956 (Wpi-RNase J) via displacement with pSC101 [71, 72] after a few culture passages by streaking the colonies on M9-glycerol plates in the presence of Tc but without Km. The colony size and growth rate were not increased by the addition of 0.1% L-(+)-arabinose after this replacement, thereby demonstrating the ability of Bsu-RNase Y, Bsu-RNase J1/J2, and Wpi-RNase J to restore the lethality due to Eco-RNase E deficiency to form colonies on solid media. The loss of the *rne* gene from these strains was confirmed by PCR (data not shown).

**Fig 3:**
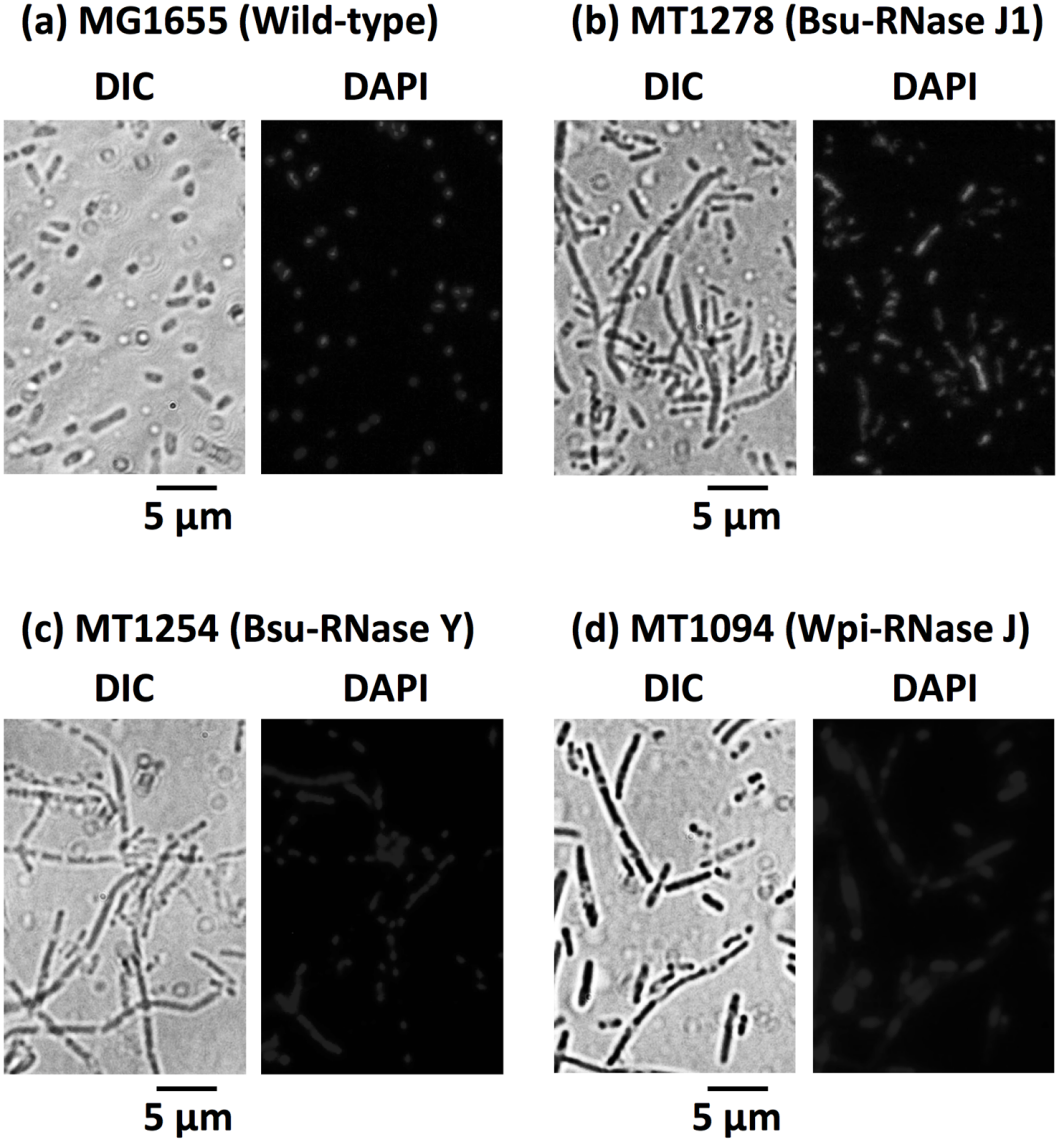
Morphology of Δ*rne E*. *coli* cells complemented by Bsu-RNase J1, Bsu-RNase Y, or Wpi-RNase J. Single colonies of the parental MG1655 strain (Wild-type) (a), MT1278 (Bsu-RNase J1) (b), MT1254 (Bsu-RNase Y) (c), and MT1094 (Wpi-RNase J) (d) grown on M9-glycerol plates (0.6% gellan gum) were suspended in M9 minimal medium and then spread directly onto glass slides. Slides were prepared for microscopic observations, as previously described [25], except that poly-L-lysine was omitted. Cells were stained with DAPI (ProLong Diamond Antifade Mountant with DAPI) and microscopic images were obtained using an Axiovert 200 system (Zeiss). A scale bar measuring 5 μm is shown below each DIC image.

More than 4 days were required to form solid colonies (approximately 1.0 mm in diameter) and the resulting Bsu-RNase Y- or RNase J-complemented Δ*rne E*. *coli* cells exhibited extensive filament formation with unevenly-distributed nucleoids, which are typical of the *E*. *coli* Δ*rne* mutation (Fig 3), thereby indicating that the ability of these enzymes to complement Eco-RNase E essentiality was conditional and only partial.

### Restoration of CFA in Δ*rne E*. *coli* by Bsu-RNase Y, Bsu-RNase J1/J2, or Wpi-RNase J has different dependencies on endogenous Eco-RNase G

**Fig 4:**
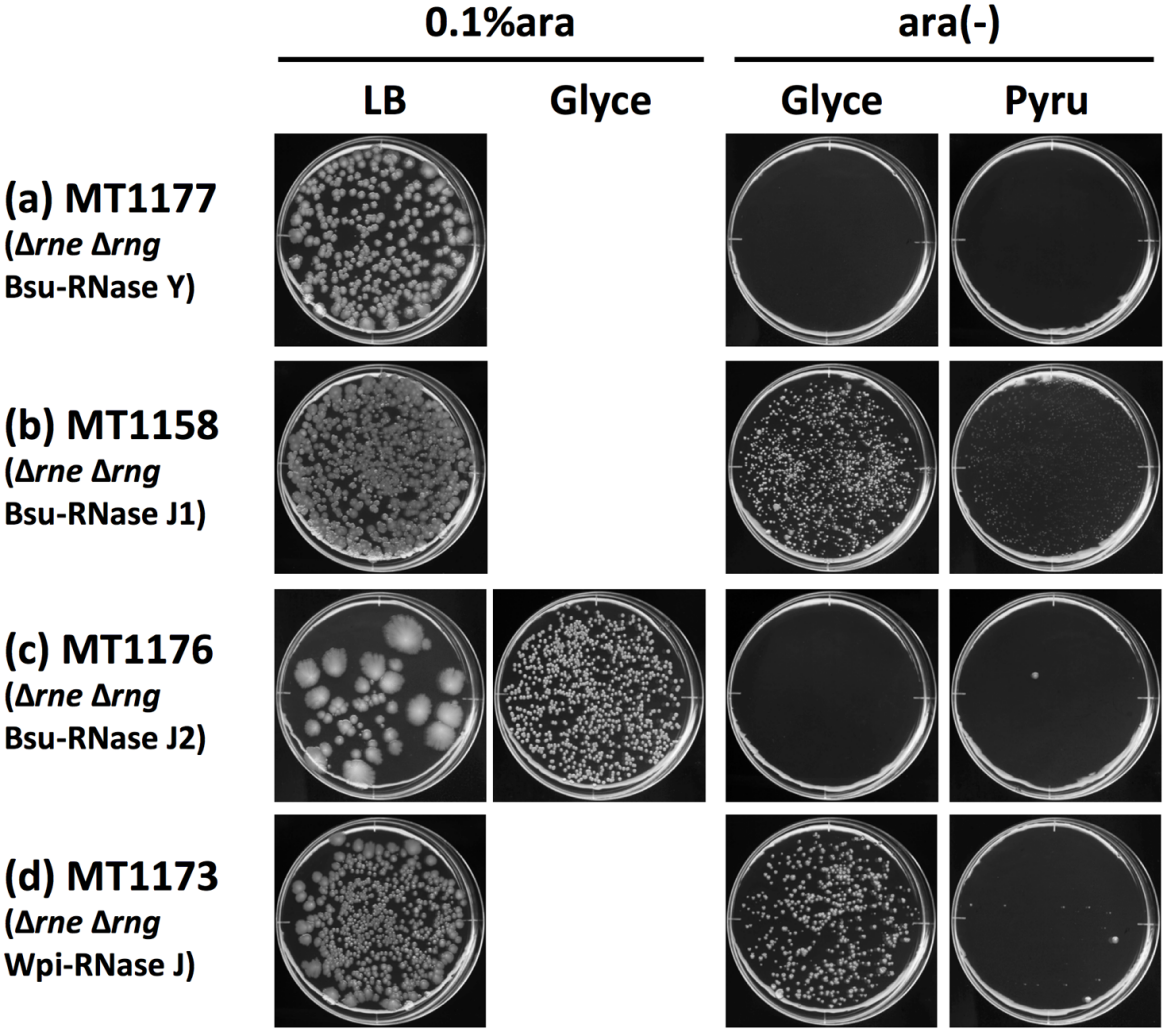
Effects of endogenous Eco-RNase G on the restoration of CFA in Bsu-RNase Y-, Bsu-RNase J1/J2-, or Wpi-RNase J-complemented Δ*rne E*. *coli*. Cultures of MT1177 (a), MT1158 (b), MT1176 (c), or MT1173 (d) were spread onto LB and M9 plates (0.6% gellan gum) with various carbon sources containing (0.1% ara) or lacking [ara(-)] 0.1% L-(+)-arabinose, as indicated. Appropriate concentrations of IPTG (10 μM for MT1173 and MT1176; 50 μM for MT1177; no IPTG (leaky expression) for MT1158) were added to the plates. Plates were scanned after incubation at 37°C for 14 days. *Glyce* glycerol, *Pyru* pyruvate.

The overproduction of Eco-RNase G and its derivatives is known to restore the growth of Δ*rne E*. *coli* [13, 24, 26], and we also found that endogenous Eco-RNase G was necessary for establishing second-site suppression of *E*. *coli* Δ*rne* lethality in N3433-based strains [52]. To investigate whether the restoration of CFA in Δ*rne E*. *coli* by Bsu-RNase Y, Bsu-RNase J1/J2, and Wpi-RNase J required the presence of endogenous Eco-RNase G, we constructed Δ*rne* Δ*rng* double mutant *E*. *coli* strains complemented by each of these RNases (MT1177, MT1158, MT1176, and MT1173) (Fig 4). In all four strains, the rate (speed) of colony formation was slower than that in Δ*rne rng*^+^ *E*. *coli* (cf. Fig 2) and incubation for 14 days was required to visualize the colonies clearly. Three different profiles of Eco-*rng* dependence were observed for the restoration of CFA. No CFA in MT1177 (Bsu-RNase Y) and MT1176 (Bsu-RNase J2) were observed either on glycerol or pyruvate media (Fig 4a and 4c), thereby indicating that endogenous Eco-*rng* is necessary to support basic sugar utilization for CFA even when Bsu-RNase Y or Bsu-RNase J2 is present. CFA in MT1158 (Bsu-RNase J1) was observed in both media conditions (Fig 4b), which indicates that the basic functions of both glycolysis and gluconeogenesis (upstream of pyruvate) were restored independently of endogenous Eco-*rng*. MT1173 (Wpi-RNase J) exhibited CFA on glycerol but not on pyruvate media (Fig 4d), thereby indicating that pyruvate utilization is dependent on endogenous Eco-*rng* but without affecting basic glycolysis. These results suggest that independent genetic factors are required for CFA with each carbon source, and that the combination of endogenous Eco-*rng* and either one of Bsu-RNase Y, Bsu-RNase J2, or Wpi-RNase J was necessary to restore CFA in all three medium conditions. It should be noted that decreased CFE was observed for MT1176 (Bsu-RNase J2) on LB but not on glycerol in the presence of 0.1% L-(+)-arabinose (Fig 4c), which cannot be explained at present. These results suggest that the requirement of Eco-RNase E for carbon utilization is multifaceted and that *E*. *coli* endogenous Eco-*rng* has essential roles in the utilization of certain carbon sources in the absence of Eco-RNase E. Our results also indicate that different RNase J homologues from different bacteria species had distinct activities in the restoration of Eco-RNase E deficiency.

### Restoration of *E*. *coli* Δ*rne* lethality by RNase J and RNase Y depend on their endonucleolytic activities

Eco-RNase E is an endoribonuclease, so we also investigated whether *E*. *coli* Δ*rne* complementation depended on the endonucleolytic activities of RNase J and RNase Y. RNase J has both endonucleolytic and 5′-to-3′ exonucleolytic activities [40, 73]. According to structural analyses, RNase J has only one catalytic center and the introduction of double mutations (D78K and H79A) into the catalytic region of Bsu-RNase J1 abolishes both its endo- and exonucleolytic activities [74]. Equivalent mutations in Wpi-RNase J are D77K and H78A according to our sequencing analysis. Bsu-RNase Y exhibits only endonucleolytic activity [50] and the introduction of double mutations (H367A and D368A) abolishes its endonucleolytic activity [49].

**Fig 5:**
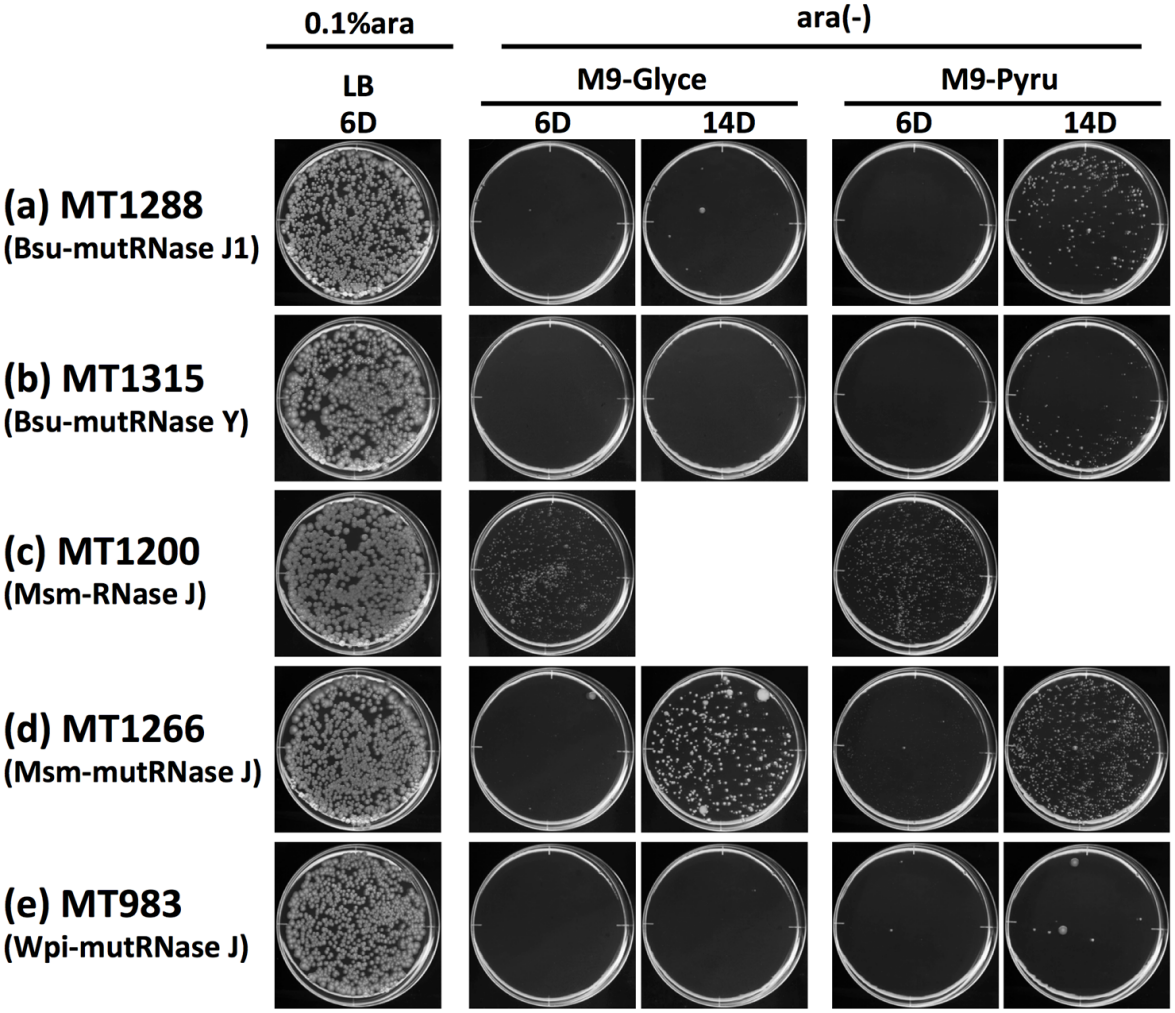
Effects of the ribonucleolytic activity on the restoration of CFA in Δ*rne E*. *coli*. Cultures of MT1288 (a), MT1315 (b), MT1200 (c), MT1266 (d), or MT983 (e) were spread onto LB or M9 plates (0.6% gellan gum) with various carbon sources containing (0.1% ara) or lacking [ara(-)] 0.1% L-(+)-arabinose, as indicated. Appropriate concentrations of IPTG (10 μM for MT983; 50 μM for MT1315; no IPTG (leaky expression) for MT1200, MT1266, and MT1288) were added to the plates. Plates were scanned after incubation at 37°C for 6 days (6D) or for 14 days (14D), as indicated. *Glyce* glycerol, *Pyru* pyruvate.

Interestingly, the introduction of double mutations (D85K and H86A) into RNase J (Msm-RNase J) from *M*. *smegmatis* strongly impaired majority of its exonucleolytic activity and considerable endonucleolytic activity was retained [46]. We consider that Msm-RNase J is a good tool for distinguishing the endonucleolytic and exonucleolytic activities, and thus we prepared plasmids that expressed the wild-type Msm-RNase J enzyme or D85K-H86A mutated enzyme (Msm-mutRNase J) for the *E*. *coli* Δ*rne* complementation assay. Disruption of both the endonucleolytic and exonucleolytic activities of Bsu-RNase J1 with D78K-H79A mutations (Bsu-mutRNase J1) eliminated its ability to restore CFA to Δ*rne E*. *coli* on glycerol or pyruvate (~15% CFE) (Fig 5a). Disruption of the endonucleolytic activity of Bsu-RNase Y with H367A-D368A mutations (Bsu-mutRNase Y) also eliminated its ability to restore CFA in Δ*rne E*. *coli* on glycerol or pyruvate (~13% CFE) (Fig 5b). In addition to Bsu-RNase J1/J2 and Bsu-RNase Y (see Fig 2), the natural form Msm-RNase J conferred CFA in Δ*rne E*. *coli* on glycerol or pyruvate after incubation for 6 days (Fig 5c). In contrast to Bsu-RNase J1 or Bsu-RNase Y, D85K-H86A mutations in Msm-RNase J did not abolish the ability to restore CFA, although the rate of colony formation slowed down (Fig 5d). Equivalent amount of RNase expression between wild-type enzyme and mutant enzyme was confirmed by SDS-PAGE analysis (S6 Fig). These results suggest that the endonucleolytic activity of RNase J and RNase Y is sufficient to restore CFA in Δ*rne E*. *coli* on glycerol or pyruvate, although the exonucleolytic activity is necessary to enhance the speed of colony formation. The D77K-H78A mutated Wpi-RNase J also lost its ability to restore CFA in Δ*rne E*. *coli* on glycerol or pyruvate (Fig 5e), thereby suggesting that Wpi-RNase J is a ribonuclease with similar enzymatic features to Bsu-RNase J1 and that the ribonucleolytic activity of Wpi-RNase J is necessary to restore the lethality due to Eco-RNase E deficiency.

### Distinction of the RNase E and RNase G activities using phenotypic defects with *E*. *coli rne rng* double mutant bacteria

**Fig 6:**
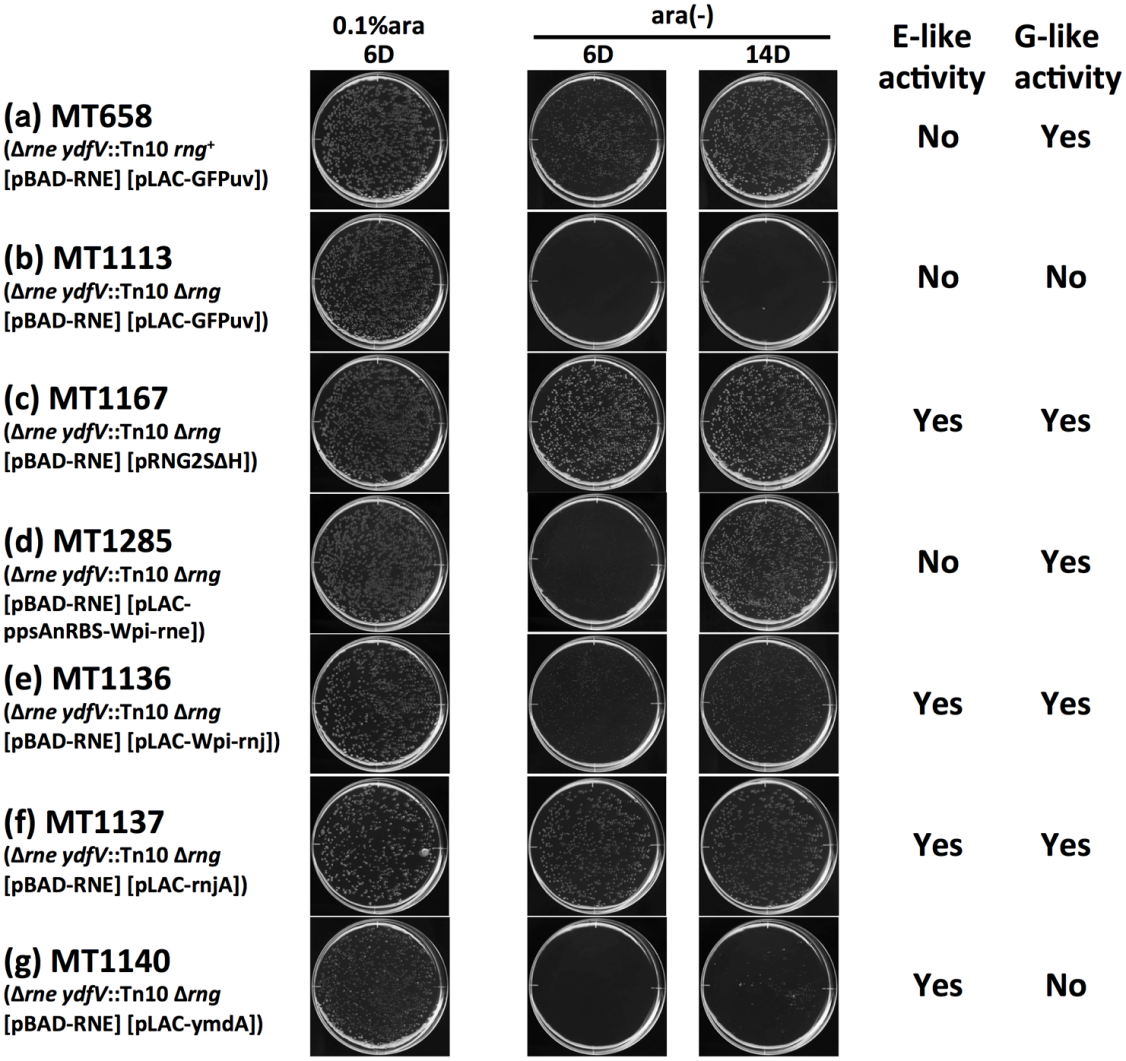
Growth of Δ*rne ydfV*::Tn10 Δ*rng E*. *coli* strains complemented with various RNases. Cultures of MT658 (a), MT1113 (b), MT1167 (c), MT1285 (d), MT1136 (e), MT1137 (f), or MT1140 (g) were spread onto M9-pyruvate plates (0.6% gellan gum) containing (0.1% ara) or lacking [ara(-)] 0.1% L-(+)-arabinose, as indicated. Appropriate concentrations of IPTG (10 μM for MT1136, MT1167, and MT1285; 50 μM for MT1140; no IPTG (leaky expression) for MT658, MT1113, and MT1137) were added to the plates. Plates were scanned after incubation at 37°C for 6 days (6D) or 14 days (14D), as indicated. A summary of the results (combined with the results in Fig 2) is shown on the right-hand side.

The length of Wpi-RNase E is 591 aa with an approximately 50-aa insertion in the S1 domain, which is typical of α-proteobacteria (see [6] for review). Considering that this insertion does not exist in the Eco-RNase E sequence, the “net” length of Wpi-RNase E is closer to that of Eco-RNase G (489 aa) rather than that of Eco-RNase E (1061 aa), although BLAST search and phylogenetic analysis detected higher similarity in the catalytic region of Eco-RNase E (also see Fig 1). Eco-*rng* is not an essential gene; thus, the function of RNase G was considered mostly covered by the RNase E function, which made it difficult to distinguish the RNase G function phenotypically from the RNase E function, although the cleavage or degradation of certain transcripts is RNase G-specific [7, 13, 75, 76]. We reported previously that *ydfV*::Tn10 mutation (second-site suppressor) allowed *E*. *coli* to grow in the absence of essential RNase E presumably by repressing the expression of RelB, the anti-toxin of RelBE anti-toxin/toxin system [52]. Experiments conducted to examine the Tn10-inserted Δ*rne E*. *coli* revertant strain (Δ*rne ydfV*::Tn10 strain) showed that endogenous Eco-*rng* was essential for growth on pyruvate minimal medium in this strain background (Fig 6b). This growth defect was not restored by adding casamino acid (0.05%) or the aa isoleucine (50 μg/ml) (Tamura et al., unpublished data), which suggests that the CFA-defective phenotype of Δ*rne ydfV*::Tn10 Δ*rng* was not caused simply by the defect in a certain aa production pathway, as shown previously [77]. However, the endogenous Eco-*rng*, which is not normally sufficient to complement *E*. *coli* Δ*rne* lethality, was sufficient to restore the CFA (Fig 6a).

We employed this Eco-*rng* null defective CFA phenotype to distinguish the RNase G-like activity by complementing various RNases of interest based on multi-copy plasmids. The presence of endogenous Eco-*rng* was sufficient to restore CFA (Fig 6a) whereas the overproduction of GFPuv, as a negative control, did not confer CFA in Δ*rne ydfV*::Tn10 Δ*rng* bacteria (Fig 6b). The overproduction of Eco-RNase G from the pNRNE4 plasmid-based gene expression system, which is known to restore *E*. *coli* Δ*rne* lethality [13, 60], restored the defective CFA obtained via Eco-*rng* null mutations, thereby confirming the validity of this assay system (Fig 6c). Wpi-RNase E, which did not restore the *E*. *coli* Δ*rne* lethality (cf. Fig 2f), restored CFA to indicate a functional relationship between Wpi-RNase E and Eco-RNase G (Fig 6d). Wpi-RNase J and Bsu-RNase J1, which restored the *E*. *coli* Δ*rne* lethality (cf. Fig 2d and 2g), also restored the CFA, which indicates that these enzymes possess the features of both Eco-RNase E and Eco-RNase G (Fig 6e and 6f). Bsu-RNase Y, which restored the *E*. *coli* Δ*rne* lethality (cf. Fig 2c), did not restore the defective CFA obtained via Eco-*rng* null mutations, thereby suggesting separate activities for Eco-RNase E and Eco-RNase G in the pyruvate utilization pathway of *E*. *coli* (Fig 6g). These results indicate that homologues of RNase E/G, RNase J, and RNase Y exhibit a variety of enzymatic features in terms of their Eco-RNase E- and Eco-RNase G-like activities.

To further evaluate the RNase E/G-like activity in RNase Y and RNase J, accumulation of 16S rRNA precursors were examined by a method described by Wachi *et al*. [8]. Overproduction of Eco-RNase E or Eco-RNase G restored the cleavage of the precursors to produce mature 16S rRNA (S7b Fig, lane 2 and 3, respectively). Overproduction of Bsu-RNase J1 or Wpi-RNase J also restored the production of 16S rRNA (S7b Fig, lane 4 and 6, respectively). Overproduction of Bsu-RNase Y or Wpi-RNase E accumulated 16.3S precursor band and produced mature 16S rRNA inefficiently (S7b Fig, lane 5 and 7, respectively). These results suggest that Bsu-RNase J1, Bsu-RNase Y, Wpi-RNase J, and Wpi-RNase E have Eco-RNase E/G-like activity sufficient to produce 16S rRNA, although the efficiency of Bsu-RNase Y or Wpi-RNase E cleavage was lower than RNase J.

## Discussion

Our results indicate that RNase J and RNase Y have the ability partially to restore the lethality due to Eco-RNase E deficiency. A similar cleavage site to Eco-RNase E was reported for Bsu-RNase J [40] and RNase Y [49, 50], and similarity of interaction partners and Eco-RNase E-like degradosome assembly was reported for Bsu-RNase Y [38, 51]. There is no published phenotypic evidence, however, for a functional relationship between these enzymes. Our results suggest that bacteria that lack RNase E homologues have a similar enzymatic activity, which is presumably endonucleolytic, to Eco-RNase E via RNase Y or RNase J, thereby indicating that an Eco-RNase E-like activity is distributed among a wider range of bacterial species than previously considered. Various types of genomic and biochemical data suggest that all bacterial species, except for some *Candidatus* strains, contain at least one RNase E, RNase G, RNase J, or RNase Y homologue [6, 50], which indicates that an Eco-RNase E-like enzymatic activity exists in all these bacteria. The exosome is the major RNA decay machinery in eukaryotes [34] but human cells also possess *ard-1* with a sequence that is not homologous to Eco-RNase E, although it can phenotypically complement *rne*^ts^ [78]. Overall, our results suggest that an enzymatic activity that establishes Eco-RNase E essentiality might be distributed widely in different classes of enzymes among living organisms ranging from bacteria to eukaryotes.

In this study, we cloned and genetically characterized Wpi-RNase E/G and Wpi-RNase J from *W*. *pipientis*. Our phylogenetic analyses based on aa sequences suggested that Wpi-RNase E/G should be categorized as an RNase E, but the complementation assays of *E*. *coli* Δ*rne* and *E*. *coli* Δ*rng* demonstrated that Wpi-RNase E is capable of complementing Eco-RNase G rather than Eco-RNase E. This result might reflect a functional shift from RNase E to RNase G, or vice versa, in an ancestor of *W*. *pipientis* (α-proteobacteria) or *E*. *coli* (γ-proteobacteria).

According to the KEGG database, we found a remarkable variety of RNase E/G lengths (344 to 1123 aa) among α-proteobacteria (S1 Table). This is an interesting observation, considering that the average gene length is generally highly conserved in prokaryotes [79]. Thus, we examined whether there was any sign of directional selection in the length of RNase E/G during the diversification of α-proteobacterial lineages. The phylogenetic tree constructed for α-proteobacterial lineages based on 16S rRNA gene sequences showed that the subclass Rickettsidae (including both Rickettsiales and Pelagibacterales) consistently had remarkably short RNase E lengths (≤ 720 aa) (S8 Fig). The phylogenetic tree based on the aa sequences of RNase E had a similar topology to that based on 16S rRNA gene sequences (S9 Fig), thereby excluding the possibility of the horizontal transfer of RNase E. The members of the subclass Caulobacteridae showed longer RNase E lengths with exceptions in two species, *R*. *elongatum* and *Ca*. Liberibacter asiaticus, which had short RNase E length (608 and 723 aa, respectively). It is considered more parsimonious to speculate that the short RNase E is a derived trait rather than an ancestral trait. However, detailed functional and structural analyses remain to be made to draw any conclusions about the evolutionary history of the length polymorphism of RNase E.

Our finding that mutations in conserved amino acids necessary to establish a ribonucleolytic activity for Bsu-RNase J1 [74] or Msm-RNase J [46] also abolished the ability of Wpi-RNase J to restore the lethality due to Eco-RNase E deficiency strongly suggests that Wpi-RNase J shares similar enzymatic characteristics with RNase J. Using mutated Msm-RNase J, we also found that the endonucleolytic activity of the enzyme was necessary to restore CFA in Δ*rne E*. *coli*, which is unsurprising considering that Eco-RNase E is an endoribonuclease. Previous studies have not reported a common cleavage site for RNase J and RNase Y. The common feature of conferring CFA in Δ*rne E*. *coli* implies that they share similar characteristics as endoribonucleases, but our results cannot exclude the possibility that bulk RNA decay rather than the specific cleavage of a target RNA might be the mechanism for restoring CFA. Although recent studies revealed the role of Hfq on processing or degradation of various RNAs by RNase E [20, 80], involvement of Hfq on RNase E essentiality is improbable, since hfq is not an essential gene in *E*. *coli* and RNase E lacking the scaffold region necessary to interact with Hfq still is able to restore CFA of Δ*rne E*. *coli*.

We developed an assay system to evaluate Eco-RNase G based on the defective growth of the *rne ydfV*::Tn10 *rng* strain on pyruvate minimal medium. Eco-RNase G is not an essential enzyme for *E*. *coli* growth and this feature made it difficult to evaluate its activity by genetic screening. Eco-RNase G is recognized as a homologue of Eco-RNase E, but the influence on certain RNAs is specific to Eco-RNase G [13, 75–77, 81]. This feature may have caused the conditional synthetic lethality only on pyruvate minimal medium in the absence of both Eco-*rne* and Eco-*rng*. Thus, using this assay, we could demonstrate that Wpi-RNase E has an Eco-RNase G-like activity, and we phenotypically distinguished the ability to complement Δ*rne* or Δ*rng* with Bsu-RNase Y for Eco-RNase G and RNase J.

We found that some medium conditions allowed RNase J and RNase Y phenotypically to restore the lethality due to Eco-RNase E deficiency in *E*. *coli*, but this restoration was limited to M9 minimal medium and did not occur on LB medium. As reported previously, CFA in Δ*rne E*. *coli* on LB is restored either by the *deaD*::Tn10 insertion mutation or by Eco-RNase G overproduction, but not by the *ydfV*::Tn10 insertion mutation [28, 60]. These results suggest that the genetic factor(s) necessary for supporting growth on LB is genetically distinct from the factor(s) required for growth on minimal medium and it is not restored by RNase J or RNase Y complementation (see Fig 2). The genetic factor(s) responsible for LB growth by *E*. *coli* (which cannot be explained by nutrient auxotrophy) remains unknown at present.

In summary, the culture of Δ*rne E*. *coli* on M9 minimal medium was used to evaluate the functional similarity between Eco-RNase E and RNase J or RNase Y from other organisms. We cloned and genetically characterized Wpi-RNase E/G as RNase G and Wpi-RNase J as RNase J. The various phenotypes observed after complementation by the ribonucleases tested in this study suggest that the normal function of Eco-RNase E is diverse during *E*. *coli* carbon source utilization. Our results indicate that the fundamental function of Eco-RNase E in conferring CFA in Δ*rne E*. *coli* is also mediated by RNase J and RNase Y in various bacterial species.

## Supporting information

S1 Fig PCR amplification and *in vivo* protein expression of Wpi-*rne* and Wpi-*rnj*. (a) PCR amplification of Wpi-*rne*, *W*. *pipientis hcpA*, bacterial 16S ribosomal DNA, and the host cell mitochondrial COI fragment. Agarose gel (1%) stained with ethidium bromide. The arrowhead indicates the expected size of the Wpi-*rne* PCR product. Lane 1, λHindIII marker (the size is shown on the left); lane 2, PCR product obtained using BmN4 as the template; lane 3, PCR product obtained using DK101-infected BmN4 as the template. (b) PCR amplification of Wpi-*rnj*. Agarose gel (1%) stained with ethidium bromide. The arrowhead indicates the expected size of the Wpi-*rnj* PCR product. Lane 1, PCR product obtained using BmN4 as the template; lane 2, PCR product using DK101-infected BmN4 as the template; lane 3, λHindIII marker. (c) SDS-PAGE analysis of Wpi-RNase E/G and Wpi-RNase J proteins. *W*. *pipientis* RNase E/G and RNase J proteins were expressed in *E*. *coli* DH5α using IPTG-inducible plasmids, i.e., pLAC-Wpi-rne and pLAC-Wpi-rnj, respectively. Bacterial cultures were grown in the presence of 50 μM IPTG, harvested, and separated on a 10% SDS polyacrylamide gel. The gel was stained with CBB. Lanes 1 and 5, protein size markers (the sizes are shown on the left side); lane 2, lysate from DH5α with pLAC-Wpi-rne (MT912); lane 3, lysate from DH5α; lane 4, lysate from DH5α with pLAC-Wpi-rnj (MT949). The asterisks indicate the expected sizes of Wpi-RNase E/G and Wpi-RNase J, respectively.
(PDF)

S2 Fig Aligned amino acid sequences of RNase E derived from 11 strains of *W*. *pipientis*. A gap is indicated with dash. Host organisms of *W*. *pipientis* are as follows. *w*CI: *E*. *mandarina*, (singly-infected), *w*Pip: *Culex pipientis*, *w*No, *w*Ri, *w*Ha and *w*Au: *Drosophila simulans*, *w*Mel: *Drosophila melanogaster*, *w*Cle: *Cimex lectularius*, *w*Bm: *Brugia malayi*, *w*Ov: *Onchocerca volvulus*, *w*Oo: *Onchocerca ochengi*.
(PDF)

S3 Fig Evaluation of appropriate IPTG concentration to visualize colonies for different RNase-complemented Δ*rne E*. *coli* strains. Cultures of MT1070 and MT1072 (a), MT1125 (b), and MT956 (c) were spread on M9 gellan gum plates with glycerol as the sole carbon source containing (0.1% ara) or lacking [ara(-)] 0.1% L-(+)-arabinose with different concentrations of IPTG as indicated. Plates were scanned after incubation at 37°C for 6 days. *Glyce* glycerol.
(PDF)

S4 Fig Confirmation of *in vivo* protein expression of GFPuv and each RNase in Δ*rne E*. *coli* by SDS-PAGE. (a) All bacterial strains were inoculated into LB medium in the presence of 0.1% L-(+)-arabinose, cultured at 37°C to mid-log phase, harvested, washed once with LB medium, and reinoculated into LB medium at an OD_600_ of 0.05 in the absence of 0.1% L-(+)-arabinose containing the appropriate antibiotics and IPTG (10 μM for MT928, MT956, and MT1125; 50 μM for MT1072; no IPTG (leaky expression) for MT696, MT1282, and MT1070). Cultures were grown for 6 h and then harvested. The amount of 0.1 OD_600_ per well was separated on a 10% SDS polyacrylamide gel. The gel was stained with CBB. The asterisk shown on the right side indicates the expected band of each protein. The size of protein marker is shown on the right side. (b) MT928 was inoculated into LB medium in the presence of 0.1% L-(+)-arabinose, cultured at 37°C to mid-log phase, harvested, washed once with LB medium, and reinoculated into LB medium at an OD_600_ of 0.05 in the absence of 0.1% L-(+)-arabinose containing the appropriate antibiotics, IPTG, and 0.1% glucose as indicated. Cultures were grown for 6 h and then harvested. The amount of 0.1 OD_600_ per well was separated on a 10% SDS polyacrylamide gel. The gel was stained with CBB. The arrowhead shown on the right side indicates the expected size (67.1 kDa) of Wpi-RNase E protein. The size of protein marker is shown on the left side.
(PDF)

S5 Fig *In vivo* protein expression and effects of GFPuv on the restoration of CFA in Δ*rne E*. *coli*. (a) SDS-PAGE analysis of GFPuv. MT696 was inoculated into LB medium in the presence of 0.1% L-(+)-arabinose, cultured at 37°C to mid-log phase, harvested, washed once with LB medium, and reinoculated into LB medium at an OD_600_ of 0.05 in the presence of each concentration of IPTG (as indicated) lacking 0.1% L-(+)-arabinose, harvested, and the amount of 0.1 OD_600_ per well was separated on a 10% SDS polyacrylamide gel. The gel was stained with CBB. The arrowhead indicates the expected size of GFPuv protein with a calculated molecular weight of 26.8 kDa. The size of protein marker is shown on the left side. (b) Culture of MT696 was spread onto LB and M9-Glyce plates (0.6% gellan gum) with various concentration of IPTG as indicated. Plates were scanned after incubation at 37°C for 6 days.
(PDF)

S6 Fig Confirmation of *in vivo* protein expression of wild-type and mutated RNases in Δ*rne E*. *coli* by SDS-PAGE. (a) All bacterial strains were inoculated into LB medium in the presence of 0.1% L-(+)-arabinose, cultured at 37°C to mid-log phase, harvested, washed once with LB medium, and reinoculated into LB medium at an OD_600_ of 0.05 in the absence of 0.1% L-(+)-arabinose containing the appropriate antibiotics and IPTG (10 μM for MT956 and MT983; 50 μM for MT1072 and MT1315; no IPTG (leaky expression) for MT696, MT1070, MT1200, MT1266, and MT1288). Cultures were grown for 6 h and then harvested. The amount of 0.1 OD_600_ per well was separated on a 10% SDS polyacrylamide gel. The gel was stained with CBB. The asterisk shown on the right side indicates the expected band of each protein. The size of protein marker is shown on the left side. (b) MT1200 was inoculated into LB medium in the presence of 0.1% L-(+)-arabinose, cultured at 37°C to mid-log phase, harvested, washed once with LB medium, and reinoculated into LB medium at an OD_600_ of 0.05 in the absence of 0.1% L-(+)-arabinose containing the appropriate antibiotics and IPTG as indicated. Cultures were grown for 6 h and then harvested. The amount of 0.1 OD_600_ per well was separated on a 10% SDS polyacrylamide gel. The gel was stained with CBB. The arrowhead shown on the right side indicates the expected size (59.6 kDa) of Msm-RNase J protein. The size of protein marker is shown on the left side. *Glc* glucose.
(PDF)

S7 Fig Effect of various RNase complementation on processing of the 16S rRNA precursors. MG1655 or MT875 was inoculated into LB medium for small-scale culture, cultured at 37°C to mid-log phase, and harvested. CM2100, MT1163, MT1173, MT1177, MT1479, MT1481, MT1483, MT1485, or MT1487 was inoculated into LB medium in the presence of 0.1% L-(+)-arabinose, cultured at 37°C to mid-log phase, harvested, washed once with LB medium, and reinoculated into LB medium in the absence of 0.1% L-(+)-arabinose containing the appropriate IPTG. Cultures were grown for 4 h and then harvested. Total RNAs extracted from each condition were analyzed as described by Wachi *et al*. [8]. (a) Total RNA (approximately 3 μg) extracted using TRIZOL reagent (Ambion) from MG1655, MT875, CM2100, or MT1163 was electrophoresed and analyzed under UV irradiation. (b) Total RNA (approximately 3 μg) extracted from MT1479, MT1481, MT1483, MT1485, MT1177, MT1173, or MT1487 was electrophoresed and analyzed under UV irradiation. *pre* precursors of 16S rRNA.
(PDF)

S8 Fig Molecular phylogenetic tree of nucleotide sequences of 16S rDNA derived from 39 α-proteobacterial lineages. The evolutionary history was inferred by using the Maximum Likelihood method (See Materials and methods for details). The percentage of trees in which the associated taxa clustered together (bootstrap values) is shown next to the branches. The tree is drawn to scale, with branch lengths measured in the number of substitutions per site. Nodes with less than 50% bootstrap support are collapsed. KEGG organism codes are given in square brackets. Amino acid lengths of RNase E are given in parentheses. Taxa with short RNase E (< 750 a.a.) are highlighted with shading.
(PDF)

S9 Fig Molecular phylogenetic tree of amino acid sequences of RNase E derived from 39 α-proteobacterial lineages. The evolutionary history was inferred by using the Maximum Likelihood method (See Materials and methods for details). The percentage of trees in which the associated taxa clustered together (bootstrap values) is shown next to the branches. The tree is drawn to scale, with branch lengths measured in the number of substitutions per site. Nodes with less than 50% bootstrap support are collapsed. KEGG organism codes are given in square brackets. Amino acid lengths of RNase E are given in parentheses. Taxa with short RNase E (< 750 a.a.) are highlighted with shading.
(PDF)

S1 Table Length of RNase E/G homologues in α-proteobacteria. (PDF)
