# Supplementary figures and images for: Enzymatic activity necessary to restore the lethality due to Escherichia coli RNase E deficiency is distributed among bacteria lacking RNase E homologues
Source: PLoS One. 2017 May 18;12(5):e0177915. doi: 10.1371/journal.pone.0177915 (PMC5436854; doi:10.1371/journal.pone.0177915)

S1 Fig.

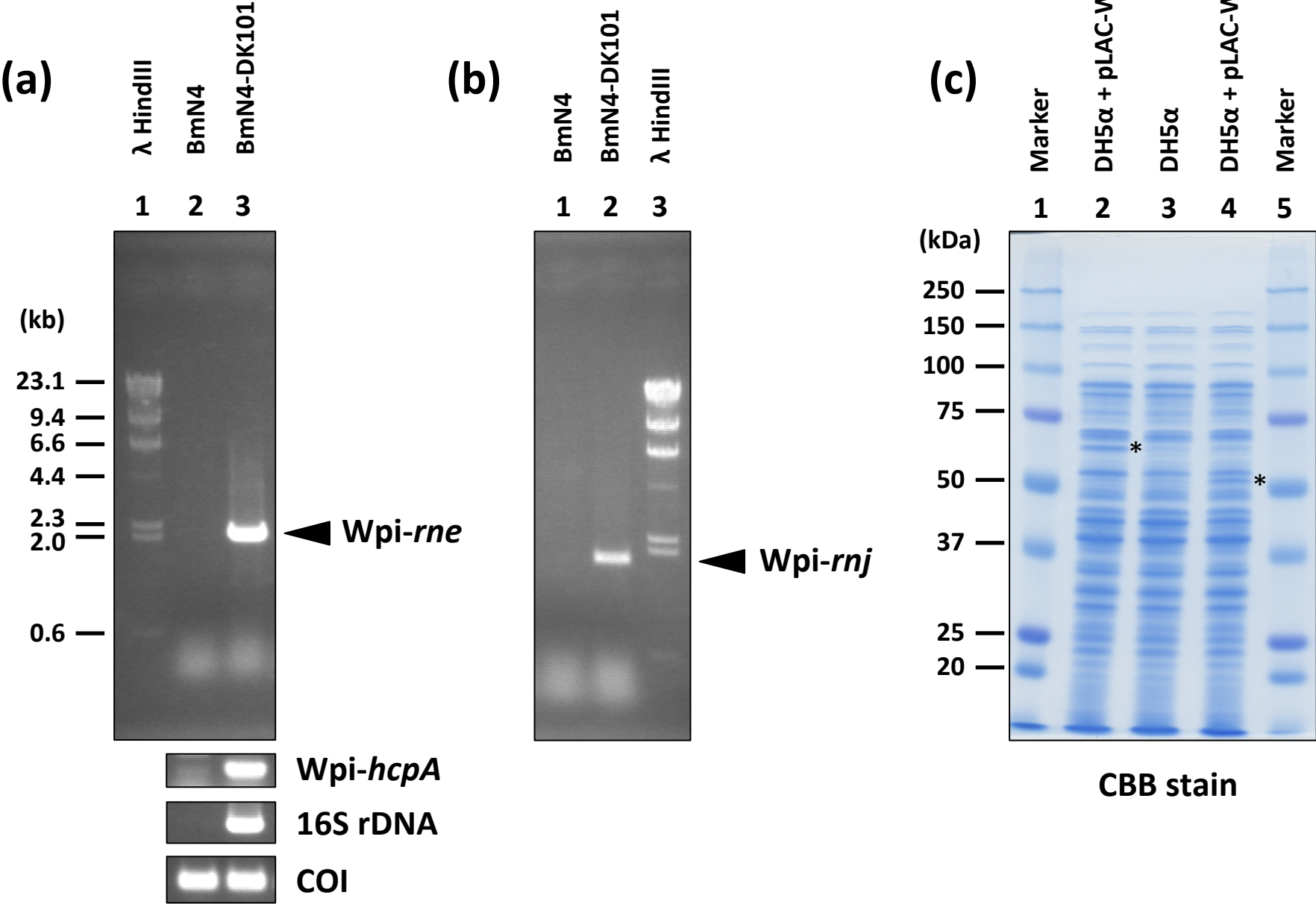

Supplement: S1 Fig — (a) PCR amplification of Wpi-rne, W. pipientis hcpA, bacterial 16S ribosomal DNA, and the host cell mitochondrial COI fragment. Agarose gel (1%) stained with ethidium bromide. The arrowhead indicates the expected size of the Wpi-rne PCR product. Lane 1, λHindIII marker (the size is shown on the left); lane 2, PCR product obtained using BmN4 as the template; lane 3, PCR product obtained using DK101-infected BmN4 as the template. (b) PCR amplification of Wpi-rnj. Agarose gel (1%) stained with ethidium bromide. The arrowhead indicates the expected size of the Wpi-rnj PCR product. Lane 1, PCR product obtained using BmN4 as the template; lane 2, PCR product using DK101-infected BmN4 as the template; lane 3, λHindIII marker. (c) SDS-PAGE analysis of Wpi-RNase E/G and Wpi-RNase J proteins. W. pipientis RNase E/G and RNase J proteins were expressed in E. coli DH5α using IPTG-inducible plasmids, i.e., pLAC-Wpi-rne and pLAC-Wpi-rnj, respectively. Bacterial cultures were grown in the presence of 50 μM IPTG, harvested, and separated on a 10% SDS polyacrylamide gel. The gel was stained with CBB. Lanes 1 and 5, protein size markers (the sizes are shown on the left side); lane 2, lysate from DH5α with pLAC-Wpi-rne (MT912); lane 3, lysate from DH5α; lane 4, lysate from DH5α with pLAC-Wpi-rnj (MT949). The asterisks indicate the expected sizes of Wpi-RNase E/G and Wpi-RNase J, respectively. (PDF) [file pone.0177915.s001.pdf]

S3 Fig.

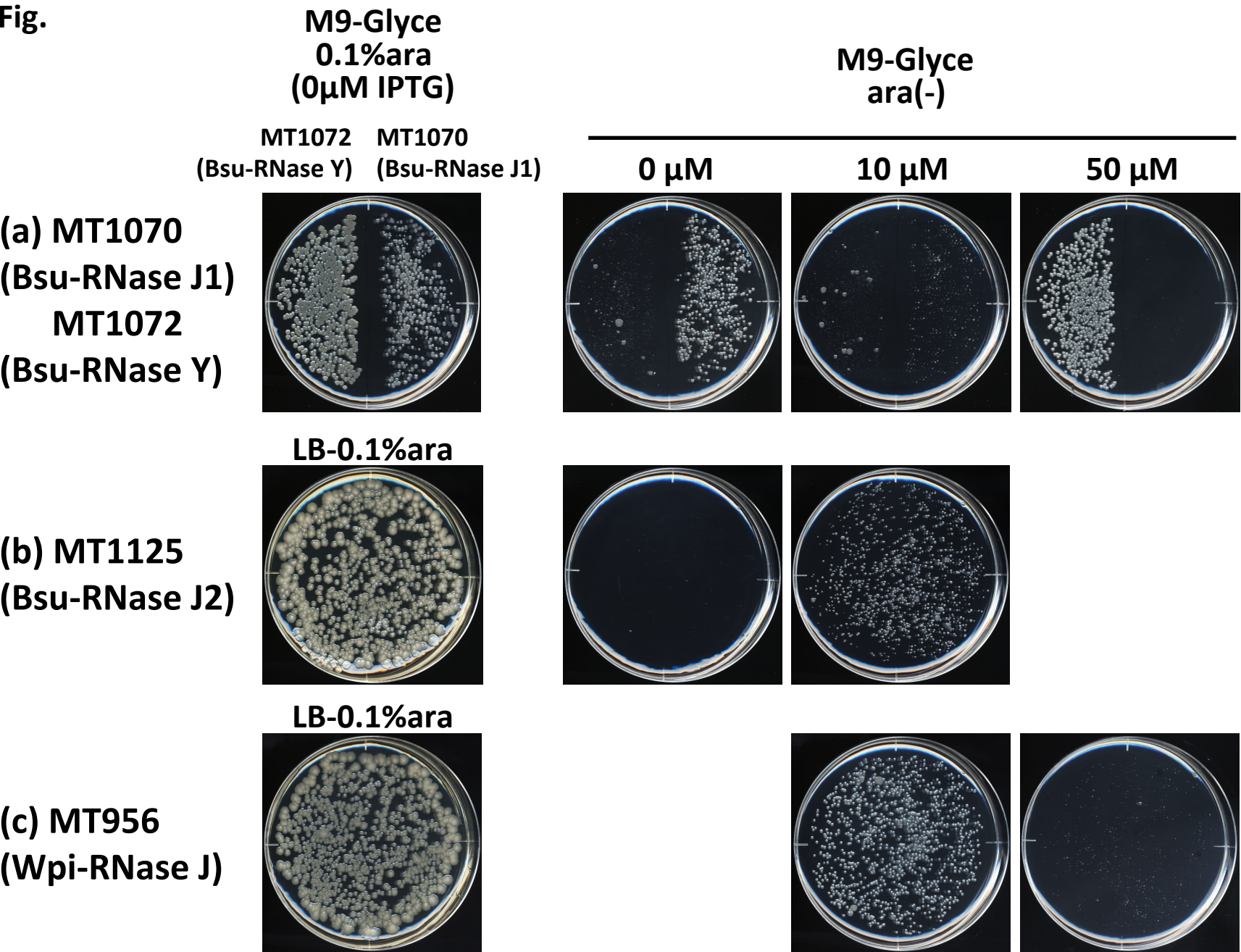

Supplement: S3 Fig — Cultures of MT1070 and MT1072 (a), MT1125 (b), and MT956 (c) were spread on M9 gellan gum plates with glycerol as the sole carbon source containing (0.1% ara) or lacking [ara(-)] 0.1% L-(+)-arabinose with different concentrations of IPTG as indicated. Plates were scanned after incubation at 37°C for 6 days. Glyce glycerol. (PDF) [file pone.0177915.s003.pdf]

S4 Fig.

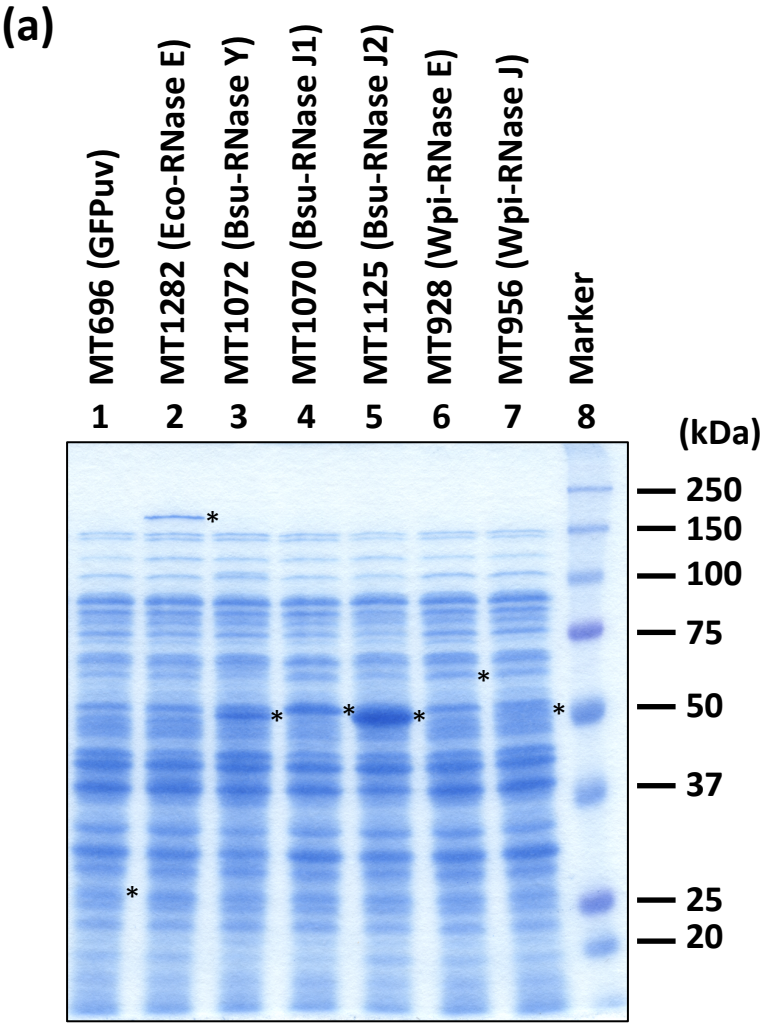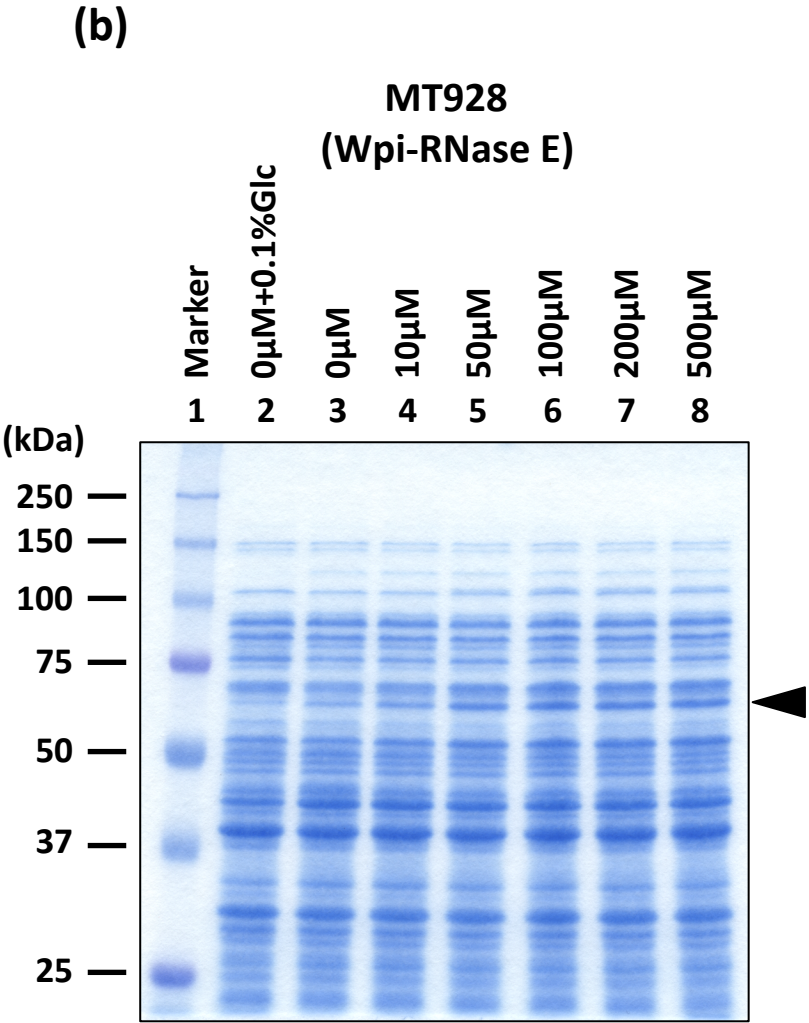

Supplement: S4 Fig — (a) All bacterial strains were inoculated into LB medium in the presence of 0.1% L-(+)-arabinose, cultured at 37°C to mid-log phase, harvested, washed once with LB medium, and reinoculated into LB medium at an OD600 of 0.05 in the absence of 0.1% L-(+)-arabinose containing the appropriate antibiotics and IPTG (10 μM for MT928, MT956, and MT1125; 50 μM for MT1072; no IPTG (leaky expression) for MT696, MT1282, and MT1070). Cultures were grown for 6 h and then harvested. The amount of 0.1 OD600 per well was separated on a 10% SDS polyacrylamide gel. The gel was stained with CBB. The asterisk shown on the right side indicates the expected band of each protein. The size of protein marker is shown on the right side. (b) MT928 was inoculated into LB medium in the presence of 0.1% L-(+)-arabinose, cultured at 37°C to mid-log phase, harvested, washed once with LB medium, and reinoculated into LB medium at an OD600 of 0.05 in the absence of 0.1% L-(+)-arabinose containing the appropriate antibiotics, IPTG, and 0.1% glucose as indicated. Cultures were grown for 6 h and then harvested. The amount of 0.1 OD600 per well was separated on a 10% SDS polyacrylamide gel. The gel was stained with CBB. The arrowhead shown on the right side indicates the expected size (67.1 kDa) of Wpi-RNase E protein. The size of protein marker is shown on the left side. (PDF) [file pone.0177915.s004.pdf]

S5 Fig.

(a)

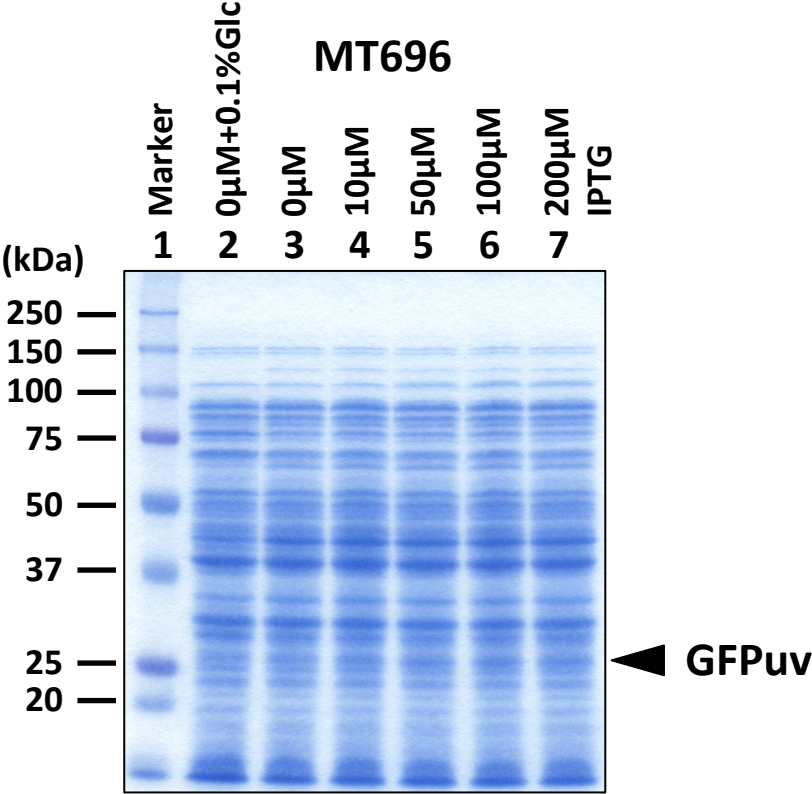

(b)

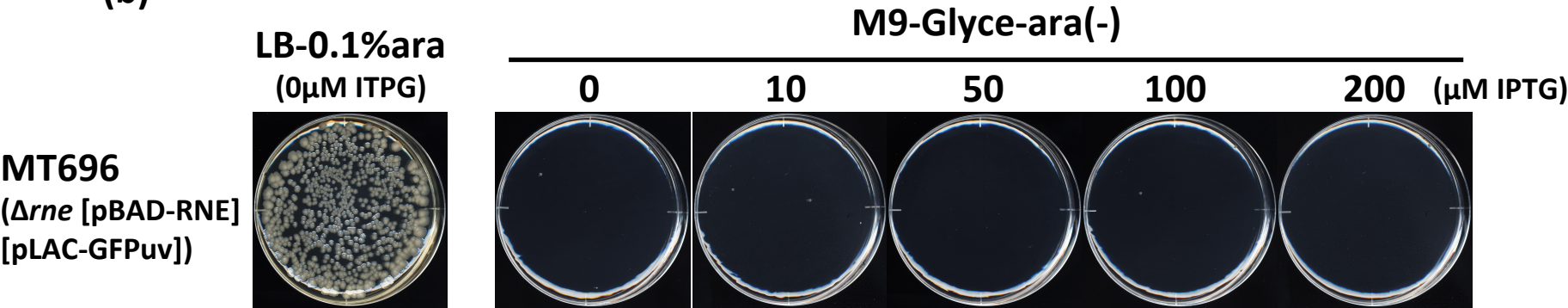

Supplement: S5 Fig — (a) SDS-PAGE analysis of GFPuv. MT696 was inoculated into LB medium in the presence of 0.1% L-(+)-arabinose, cultured at 37°C to mid-log phase, harvested, washed once with LB medium, and reinoculated into LB medium at an OD600 of 0.05 in the presence of each concentration of IPTG (as indicated) lacking 0.1% L-(+)-arabinose, harvested, and the amount of 0.1 OD600 per well was separated on a 10% SDS polyacrylamide gel. The gel was stained with CBB. The arrowhead indicates the expected size of GFPuv protein with a calculated molecular weight of 26.8 kDa. The size of protein marker is shown on the left side. (b) Culture of MT696 was spread onto LB and M9-Glyce plates (0.6% gellan gum) with various concentration of IPTG as indicated. Plates were scanned after incubation at 37°C for 6 days. (PDF) [file pone.0177915.s005.pdf]

S6 Fig.

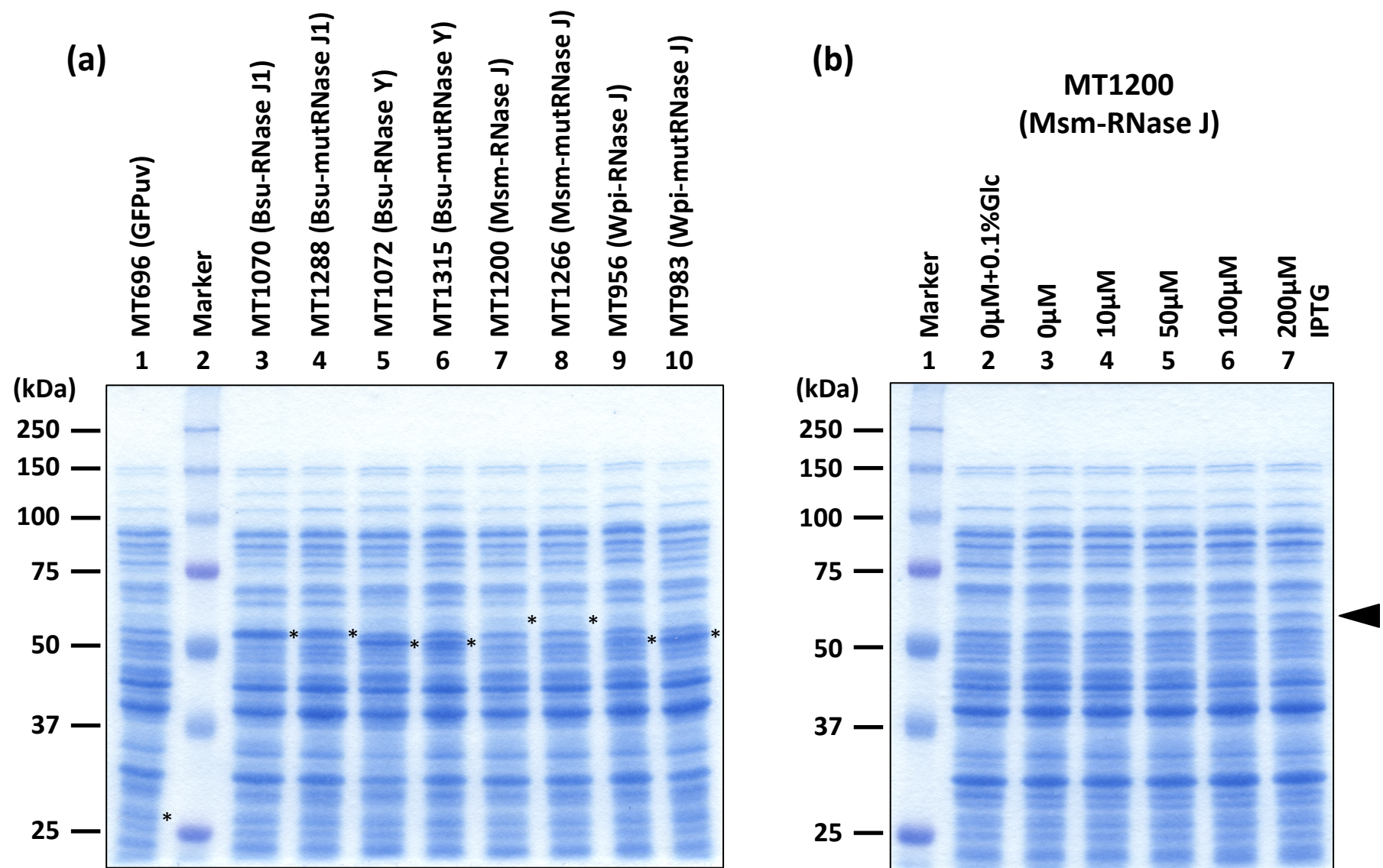

Supplement: S6 Fig — (a) All bacterial strains were inoculated into LB medium in the presence of 0.1% L-(+)-arabinose, cultured at 37°C to mid-log phase, harvested, washed once with LB medium, and reinoculated into LB medium at an OD600 of 0.05 in the absence of 0.1% L-(+)-arabinose containing the appropriate antibiotics and IPTG (10 μM for MT956 and MT983; 50 μM for MT1072 and MT1315; no IPTG (leaky expression) for MT696, MT1070, MT1200, MT1266, and MT1288). Cultures were grown for 6 h and then harvested. The amount of 0.1 OD600 per well was separated on a 10% SDS polyacrylamide gel. The gel was stained with CBB. The asterisk shown on the right side indicates the expected band of each protein. The size of protein marker is shown on the left side. (b) MT1200 was inoculated into LB medium in the presence of 0.1% L-(+)-arabinose, cultured at 37°C to mid-log phase, harvested, washed once with LB medium, and reinoculated into LB medium at an OD600 of 0.05 in the absence of 0.1% L-(+)-arabinose containing the appropriate antibiotics and IPTG as indicated. Cultures were grown for 6 h and then harvested. The amount of 0.1 OD600 per well was separated on a 10% SDS polyacrylamide gel. The gel was stained with CBB. The arrowhead shown on the right side indicates the expected size (59.6 kDa) of Msm-RNase J protein. The size of protein marker is shown on the left side. Glc glucose. (PDF) [file pone.0177915.s006.pdf]

S7 Fig.

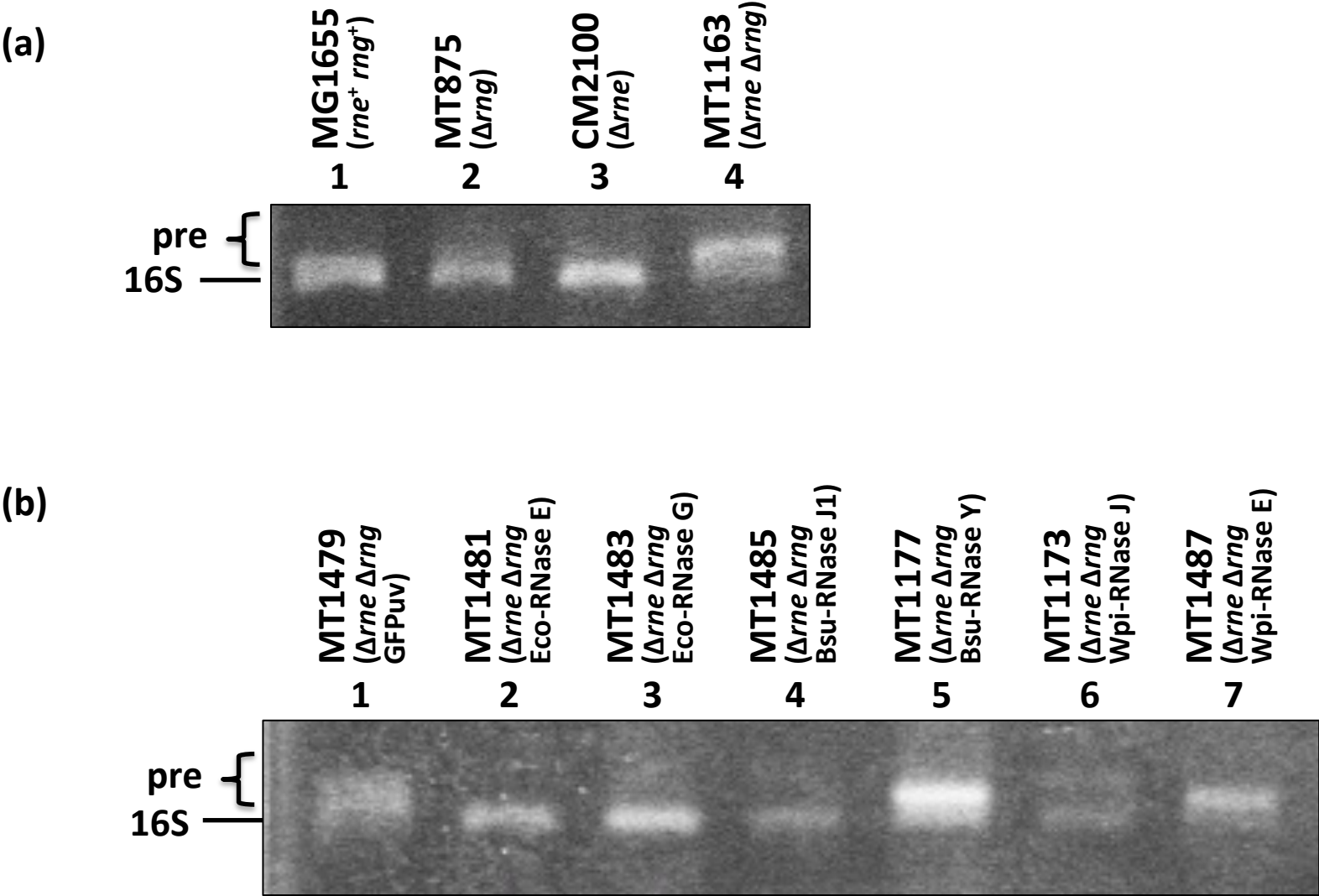

Supplement: S7 Fig — MG1655 or MT875 was inoculated into LB medium for small-scale culture, cultured at 37°C to mid-log phase, and harvested. CM2100, MT1163, MT1173, MT1177, MT1479, MT1481, MT1483, MT1485, or MT1487 was inoculated into LB medium in the presence of 0.1% L-(+)-arabinose, cultured at 37°C to mid-log phase, harvested, washed once with LB medium, and reinoculated into LB medium in the absence of 0.1% L-(+)-arabinose containing the appropriate IPTG. Cultures were grown for 4 h and then harvested. Total RNAs extracted from each condition were analyzed as described by Wachi et al. [8]. (a) Total RNA (approximately 3 μg) extracted using TRIZOL reagent (Ambion) from MG1655, MT875, CM2100, or MT1163 was electrophoresed and analyzed under UV irradiation. (b) Total RNA (approximately 3 μg) extracted from MT1479, MT1481, MT1483, MT1485, MT1177, MT1173, or MT1487 was electrophoresed and analyzed under UV irradiation. pre precursors of 16S rRNA. (PDF) [file pone.0177915.s007.pdf]

S8 Fig.

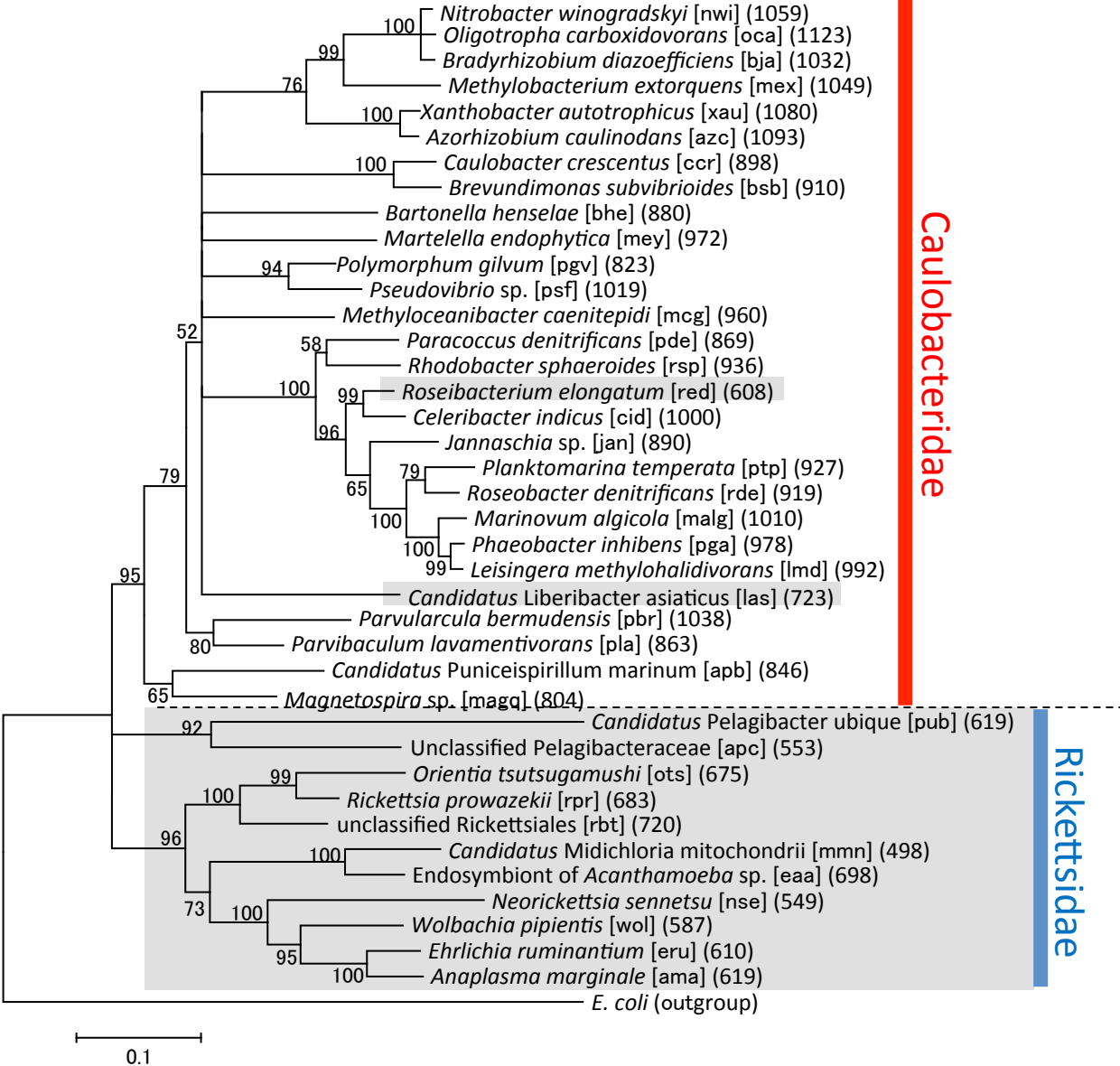

Supplement: S8 Fig — The evolutionary history was inferred by using the Maximum Likelihood method (See Materials and methods for details). The percentage of trees in which the associated taxa clustered together (bootstrap values) is shown next to the branches. The tree is drawn to scale, with branch lengths measured in the number of substitutions per site. Nodes with less than 50% bootstrap support are collapsed. KEGG organism codes are given in square brackets. Amino acid lengths of RNase E are given in parentheses. Taxa with short RNase E (< 750 a.a.) are highlighted with shading. (PDF) [file pone.0177915.s008.pdf]

S9 Fig.

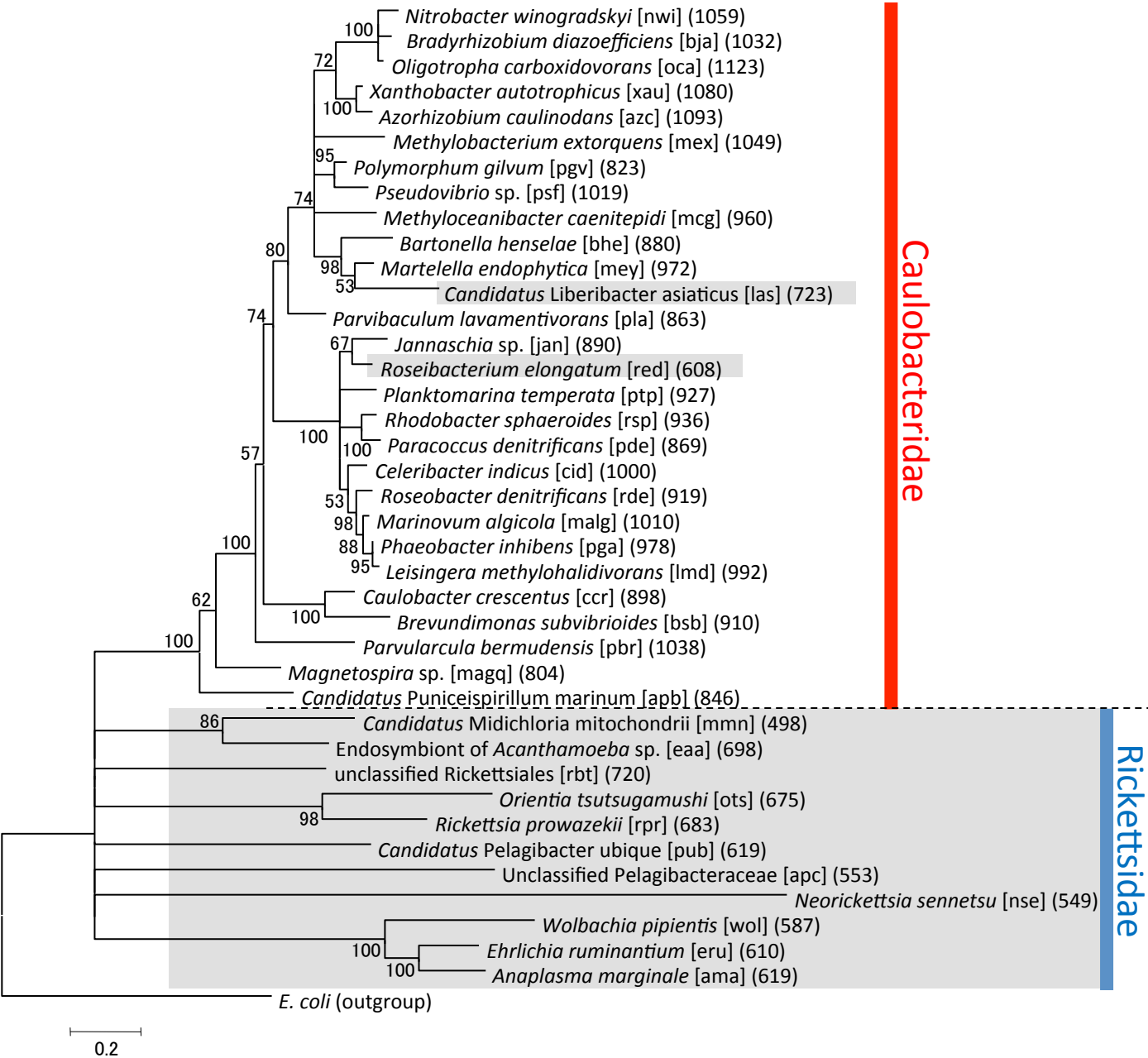

Supplement: S9 Fig — The evolutionary history was inferred by using the Maximum Likelihood method (See Materials and methods for details). The percentage of trees in which the associated taxa clustered together (bootstrap values) is shown next to the branches. The tree is drawn to scale, with branch lengths measured in the number of substitutions per site. Nodes with less than 50% bootstrap support are collapsed. KEGG organism codes are given in square brackets. Amino acid lengths of RNase E are given in parentheses. Taxa with short RNase E (< 750 a.a.) are highlighted with shading. (PDF) [file pone.0177915.s009.pdf]
