# Supplementary material for: Enzymatic activity necessary to restore the lethality due to Escherichia coli RNase E deficiency is distributed among bacteria lacking RNase E homologues
Source: PLoS One. 2017 May 18;12(5):e0177915. doi: 10.1371/journal.pone.0177915 (PMC5436854; doi:10.1371/journal.pone.0177915)
Supplement: S2 Fig — A gap is indicated with dash. Host organisms of W. pipientis are as follows. wCI: E. mandarina, (singly-infected), wPip: Culex pipientis, wNo, wRi, wHa and wAu: Drosophila simulans, wMel: Drosophila melanogaster, wCle: Cimex lectularius, wBm: Brugia malayi, wOv: Onchocerca volvulus, wOo: Onchocerca ochengi. (PDF) [file pone.0177915.s002.pdf]

S2 Fig.

```
                10        20        30        40        50        60        70        80        90       100
        ....|....|....|....|....|....|....|....|....|....|....|....|....|....|....|....|....|....|....|....|
wCI     MVSDGKRLLLIESTTCSNEVRVALLVNGRVVEFEQEFKEKKQLRGNIYVAYVKRIEPALQAVFIEYGKNKQGFLSFSEISLNYFNIPEKEKE-IFPDEDY
wPip    MVSDGKRLLLIESTTCSNEVRVALLVNGRVVEFEQEFKEKKQLRGNIYVAYVKRIEPALQAVFIEYGKNKQGFLSFSEISLNYFNIPEKEKE-IFPDEDY
wNo     MASDGKRLLLIESTTCSNEVRVALLVNDRVVEFEQEFKEKRQLRGNIYVAYIKRIEPALQAVFIEYGKNKQGFLSFSEISLNYFNIPEKEKE-IFPDEGY
wRi     MVNSGKRLLLIENSTCSNEVRVALSVNNKVVEFEQEFKEKKQLRGNIYVAYIKRIEPSLQAVFIEYGKNKQGFLSFSEISLDYFNIPEKEKETIFESYSN
wHa     MVNSGKRLLLIENSTCSNEVRVALSVNNKVVEFEQEFKEKKQLRGNIYVAYIKRIEPSLQAVFIEYGKNKQGFLSFSEISLDYFNIPEKEKETIFESYSN
wAu     MTNSGKRVLLIENSICSNEVRVALSINNKVVEFEQEFKEKKQLRGNIYVAYIKRIEPSLQAVFIEYGKNKQGFLSFSEISLDYFNIPEKEKETIFEGYSN
wMel    MTNSGKRVLLIENSICSNEVRVALSINNKVVEFEQEFKEKKQLRGNIYVAYIKRIEPSLQAVFIEYGKNKQGFLSFSEISLDYFNIPEKEKETIFEGYSN
wCle    MANSGKRLLLIENSICSDEVRVALSVNNRVVEFEQEFKEKRQLRGNIYIAHVKRVEPSLQAVFIEYGKNKQGFLSFSEISPDYFNISEKEKEAFFESYSS
wBm     MANSGKRLLLIENSICSDEVRVALSADNRVVEFEQEFKEKKQLRGNIYIACVKRVEPSLQAVFIEYGKNKQGFLSFSEISPDYFNIPEKEKETFFEGCLN
wOv     MVNNSKRLLLIENSICSDEVRVALSVNNKVVEFEQEFKEKRQLRGNIYVAYVKRIKPSLQAVFIEYGKNKQGFLSFSEISPDYFNISEKEKEAFFESCSN
wOo     MVNNSKRLLLIENSICSDEVRVALSVNNKIVEFEQEFKEKRQLRGNIYVAYVKRIKPSLQAVFIEYGKNKQGFLSFSEISPDYFNISEKEKEAFFESCSN

               110       120       130       140       150       160       170       180       190       200
        ....|....|....|....|....|....|....|....|....|....|....|....|....|....|....|....|....|....|....|....|
wCI     SSGVDCSNDNCTEEGLDSTAS---NESSSGFVREVPLHRKYKLQDVISVNQKLLVQLTKEERGNKGASFTTYVTLVGRYCVFMPNSISRGGVSRRIEDTN
wPip    SSGVDCSNDNCTEEGLDSTAS---NESSSGFVRGVPLHRKYKLQDVISVNQKLLVQLTKEERGNKGASFTTYVTLVGRYCVFMPNSISRGGVSRRIEDTN
wNo     SSGVDCSNDNCTEEGLDSTAS---NESSSGFVREVPLHRKYKLQDVISVNQKLLVQLTKEERGNKGASFTTYVTLVGRYCVFMPNSISRGGVSRRIEDTN
wRi     DDYTEKGNDTIANVSSDSAASKNVNESGSGFVREVPLYKKYKLQDVISVNQKVLVQLTKEERGNKGASFTTYITLVGRYCVFMPNSMSKGGVSRRIEDAN
wHa     DDYTEKGNDTIANVSSDSAASKNVNESGSGFVREVPLYKKYKLQDVISVNQKVLVQLTKEERGNKGASFTTYITLVGRYCVFMPNSMSKGGVSRRIEDAN
wAu     DDCIEEAS---ANVSSDSAASKNVNGSGSGFVREVPLYKKYKLQDVISVNQKVLVQLTKEERGNKGASFTTYITLVGRYCVFMPNSMSKGGVSRRIEDAN
wMel    DDCIEEAS---ANVSSDSAASKNVNGSGSGFVREVPLYKKYKLQDVISVNQKVLVQLTKEERGNKGASFTTYITLVGRYCVFMPNSMSKGGVSRRIEDAN
wCle    GDCTEEGNGASANTSSDSTTSRSVNESGNGFVREVSLYRRYKLQDVISVNQKILVQLTKEERGNKGASFTTYITLVGRYCVFMPNSMSRGGVSRRIEDIN
wBm     DDCTEENGNASANISSDSLTNKNVNESGNGFVREVSLYKRYKLQDVISVNQRLLVQLTKEERGNKGASFTTYITLVGRYCVFMPNSMSKGGVSRRIEDVN
wOv     GDYTEENSSVSTNVSSDSAANKNINESSSSFVREVSLYKKYKLQDVIAVNQKILVQLTKEERGNKGASFTTYITLVGRYCIFMPNSMSKGGISRKIEDAN
wOo     GDYTEENSSVSTNVSSDSAANKNINESSSSFVREVSLYKKYKLQDVIAVNQKILVQLTKEERGNKGASFTTYITLVGRYCIFMPNSMSKGGISRKIEDAN

               210       220       230       240       250       260       270       280       290       300
        ....|....|....|....|....|....|....|....|....|....|....|....|....|....|....|....|....|....|....|....|
wCI     VRKQLKDILNSINLPKGSGLIVRTVGAGKSKKEIEQDYNNLTLLWQNIQQNFSSLNVPSLIYNEADVVMRSVRDFCSNDTEIIVSDREVCEAVRKYTKNV
wPip    VRKQLKDILNSINLPKGSGLIVRTVGAGKSKKEIEQDYNNLTLLWQNIQQNFSSLNVPSLIYNEADVVMRSVRDFCSNDTEIIVSDREVCEAVRKYTKNV
wNo     VRKQLKDILDSINLPKSSGLIVRTVGAGKSKKEIEQDYNNLTLLWQNIQQNFSSLNVPSLIYNEADVVMRSVRDFCSNDTEIIVSDREVCEAVRKYTKNV
wRi     VRKQLKEILSSINLPKRSGLIVRTVGSGKNKKEIEQDYNYLSSLWQSIQKNAFSVNVPSLIYNEADVIMRSVRDFCSDGIEVIVSGKEAFEAVRQYANNA
wHa     VRKQLKEILSSINLPKRSGLIVRTVGSGKNKKEIEQDYNYLSSLWQSIQKNAFSVNVPSLIYNEADVIMRSVRDFCSDGIEVIVSGKEAFEAVRQYANNA
wAu     VRKQLKEILSSINLPKRSGLIVRTVGSGKNKKEIEQDYNYLSSLWQSIQKNAFSVNVPSLIYNEADVIMRSVRDFCSDGIEVIVSGKEAFEAVRQYANNA
wMel    VRKQLKEILSSINLPKRSGLIVRTVGSGKNKKEIEQDYNYLSSLWQSIQKNAFSVNVPSLIYNEADVIMRSVRDFCSDGIEVIVSGKEAFEAVRQYANNA
wCle    VRKQLKDILNSIELPKRSGLIVRTVGSGKSKKEIEQDYNYLSSLWQNIQENVSSVNVPSLIYNEADLIMKSIRDFCSNDVEVVISGKEAFETVRQYARNV
wBm     VRKQLKDILSSMDLPKRSGLIIRTIGSGKSKKEIEQDYNYLSSLWQNIQKNASSINVPSLIYNEADLIMRSIRDFCSNGMEVIVSGKEAFETVRQYARNA
wOv     IRKQLKDILDSINLPKKSCLIIRTIGSGKSKKEIEQDYNYLFSLWQNIQKNASSTNVPSLIYNEADLIMRSIRDFCSNDVEIIVSGKEAFEVVKQYTENA
wOo     IRKQLKDILDSISLPKKSCLIIRTIGSGKSKKEIEQDYNYLFSLWQNIQKNASSTNVPSLIYNEADLIMRSIRDFCSNDVEIIVSGKEAFEVVKQYTENA

               310       320       330       340       350       360       370       380       390       400
        ....|....|....|....|....|....|....|....|....|....|....|....|....|....|....|....|....|....|....|....|
wCI     LQG-KLRPRLYRGSVPIFTYYRVEDQISELYSNRVELPSGGSLVITLTEAFVSIDVNSGKMTGEDNIEETAYRTNMEALSEISRQANLRGLSGLIVVDFI
wPip    LQG-KLRPRLYRGSVPIFTYYRVEDQISELYSNRVELPSGGSLVITLTEAFVSIDVNSGKMTGEDNIEETAYRTNMEALSEISRQANLRGLSGLIVVDFI
wNo     LQG-KLRPRLYRGSVPIFTYYRVEDQISELYSNRVELPSGGSLVITLTEAFVSIDVNSGKMTGEDNIEETAYRTNMEALSEISRQANLRGLSGLIVVDFI
wRi     LKGSKLRYRLYRGYAPIFTYYGIEDQISELYSNRVELPSGGSLIITLTEAFVSIDVNSGKMTGEDSIEETAYRTNMEAVPEISRQVNLRGLSGLIVVDFI
wHa     LKGSKLRYRLYRGYAPIFTYYGIEDQISELYSNRVELPSGGSLIITLTEAFVSIDVNSGKMTGEDSIEETAYRTNMEAVPEISRQVNLRGLSGLIVVDFI
wAu     LKGSKLRYRLYRGYAPIFTYYGIEDQISELYSNRVELPSGGSLIITLTEAFVSIDVNSGKMTGEDSIEETAYRTNMEAVPEISRQVNLRGLSGLIVVDFI
wMel    LKGSKLCYRLYRGYAPIFTYYGIEDQISELYSNRVELPSGGSLIITLTEAFVSIDVNSGKMTGEDSIEETAYRTNMEAVPEISRQVNLRGLSGLIVVDFI
wCle    LKGSKLRYRLYRGFVPIFTYYGIEDQISELYSNRVKLPSGGSLIITLTEAFVSIDVNSGKMTGEDSIEETAYRTNMEAAPEISRQVNLRGLSGLIVVDFI
wBm     LKGSKLRYRLYRGFIPIFTYYGVEDQISELYSNRVKLPSGGSLIITLTEAFVSIDVNSGKMTGEDSIEETAYRTNMEAVPEISRQVNLRGLSGLIVVDFI
wOv     LKGSKLRYRLYRGFVPIFTYYGIEDQVSELYSNRVKLPSGGSLIITLTEAFVSIDVNSGKMMEEDSIEETAYRTNMEAVPEISRQVNLRGLSGLIVVDFI
wOo     LKGSKLRYRLYRGFVPIFTYYGIEDQVSELYSNRVKLPSGGSLIITLTEAFVSIDVNSGKMMEEDSIEETAYRTNMEAVPEISRQVNLRGLSGLIVVDFI

               410       420       430       440       450       460       470       480       490       500
        ....|....|....|....|....|....|....|....|....|....|....|....|....|....|....|....|....|....|....|....|
wCI     DMLKLEYCKNVEFAIKQAFKDDKAKVQFSFINDFGLMVFSRQRIKPNIQEINTTECLRCKGIGRVKSNEVVVASILRDLQHIANKNQNKSFDLIARSAVI
wPip    DMLKLEYCKNVEFAIKQAFKDDKAKVQFSFINDFGLMVFSRQRIKPNIQEINTTECLHCKGIGRVKSNEVVVASILRDLQHIANKNQNKSFDLIARSAVI
wNo     DMLKLEYCKNVEFAIKQAFKDDRAKVQFSFINDFGLMVFSRQRIKPNIQEINTTECLRCKGIGRVKSNEVVVASILRDLQHIANKNQNKSFDLIARSAVI
wRi     DMLKYQYCRAVESAIKQAFKDDKAKVQFSYINDFGLMVFSRQRIKPNIQEINTTECLHCKGTGRVKSNEVIVSSILRDLQHIANKNRNKSFDLVAHSAVI
wHa     DMLKYQYCRAVESAIKQAFKDDKAKVQFSYINDFGLMVFSRQRIKPNIQEINTTECLHCKGTGRVKSNEVIVSSILRDLQHIANKNRNKFFDLVAHSAVI
wAu     DMLKYQYCRAVESAIKQAFKDDKAKVQFSYINDFGLMVFSRQRIKPNIQEINTTECLHCKGTGRVKSNEVIVSSILRDLQHIANKNRNKSFDLVAHSAVI
wMel    DMLKYQYCRAVESAIKQAFKDDKAKVQFSYINDFGLMVFSRQRIKPNIQEINTTECLHCKGTGRVKSNEVIVSSILRDLQHIANKNRNKSFDLVAHSAVI
wCle    DMLKYQYCRAVESAIRQAFKDDKARVQFSYINDFGLMVFSRQRIKPNIQEINTTECLHCKGIGKVKSNEVIVASILRDLQYVAIRNRNRSFDLVAHGSII
wBm     DMLRYQYCRAVESAIRQAFKDDKARVQFSYINDFGLMVFSRQRIKPSIQEINTTECLHCKGIGKVKSNEVIVASILRDLQHIASRNRNRSFDLVAHGAII
wOv     GMLKYQYCRAVEFAVRQAFKDDKAKVQFSYINDFGLMVFSRQRIKPSIQEINTIECLHCRGVGKVKSNEVIVASVLRELQYVASRNRNGSFDLLAHSSII
wOo     GMLKYQYCRAVEFAVRQAFKDDKAKVQFSYINDFGLMVFSRQRIKPSIQEINTIECLHCRGVGKVKSNEVIVASVLRELQYVASRNRNGSFDLLAHSSII

               510       520       530       540       550       560       570       580       590       600
        ....|....|....|....|....|....|....|....|....|....|....|....|....|....|....|....|....|....|....|....|
wCI     AHIFNNKRDMVSTIEKEFNITLNVSIDNSLDADTFILKQGDHFSVRRCTHIINFNDYKPLQNSGCQIVNSRDKEAG---KLPGNFWLTKWLSRLLGSNN*
wPip    AHIFNNKRDMVSTIEKEFNITLNVSIDNSLDADTFILKQGDH---------INFNDYKPLQNSGCQIVNSRDKEAG---KLPGNFWLTKWLSRLLGSNN*
wNo     AHIFNNKRDMVSTIEKEFNITLNVSIDNSLDADTFILKQGDH---------VNFNDYKPLQNSGCQIVNSRDKEAGSSDKLPGNFWLTKWLSRLLSSNN*
wRi     AHIFNNKRNTVSTIEKEFNITLNVSIDNSLDVNTFVLKQGDD---------VNLNGYKPLQNSGYQVGDSSNEEADNSNKLRGNFWLTKWLSRLLSSNN*
wHa     AHIFNNKRNTVSTIEKEFNITLNVSIDNSLDVNTFVLKQGDD---------VNLNGYKPLQNSGYQIGDSSNEEADNSNKLRGNFWLTKWLSRLLSSNN*
wAu     AHIFNNKRNTVSTIEKEFNITLNVSIDNSLDVNTFVLKQGDD---------VNLNGYKPLQNSGYQIGDSSNEEADNSNKLRGNFWLTKWLSRLLSSNN*
wMel    AHIFNNKRNTVSTIEKEFNITLNVSIDNSLDVNTFVLKQGDD---------VNLNGYKPLQNSGYQIGDSSNEEADNSNKLRGNFWLTKWLSRLLSSNN*
wCle    AHIFNNKRDTVSTIEREFNIKLNVSADSNLSIDAFILKRGDD---------VNLNDCKPLQNSGYKIETSNNERADNSGGLQSNFWLTKWLSRLLSSNS*
wBm     AHIFNNKRGTVSTIEKEFNIKLNVSADSSLSVDTFVLKRGDE---------VNLSDCKPLQNSGYKIESSSNERADNSEKLQGSFWLTKWLSRILNSNS*
wOv     THIFNNKRDTVSTIEKEFNIKLNVNADNNLSVDAFILKRGDN---------VNLNDCKPLQNSGYKTEGNDNEKVNYSGGLQNNFWLTRWLSRLLSSNS*
wOo     THIFNNKRDTVSTIEKEFNIKLNVNADNNLSVDAFILKRGDN---------VNLNDCKPLQNSGYKTEGNDNEKVNYSGGLQNNFWLTRWLSRLLSSNS*
```
