# Supplementary material for: Enzymatic activity necessary to restore the lethality due to Escherichia coli RNase E deficiency is distributed among bacteria lacking RNase E homologues
Source: PLoS One. 2017 May 18;12(5):e0177915. doi: 10.1371/journal.pone.0177915 (PMC5436854; doi:10.1371/journal.pone.0177915)
Supplement: S1 Table — (PDF) [file pone.0177915.s010.pdf]

S1 Table. Length of RNase E/G homologues in α-proteobacteria.

Organisms in α-proteobacteria (from KEGG database)	RNase E/G family(aa)	KEGG Entry	RNase E/G family(aa)	KEGG Entry	GWW motif
mag Magnetospirillum magneticum	344	amb3337			No
mmn Candidatus Midichloria mitochondrii	498	midi_01107			No
nse Neorickettsia sennetsu	549	NSE_0120			No
apc Alpha proteobacterium HIMB59	553	HIMB59_00010520	375	HIMB59_00008540	No
wol Wolbachia wMel	587	WD0929			No
red Roseibacterium elongatum	608	roselon_02582	248	roselon_02583	No (608), Yes (248)
eru Ehrlichia ruminantium Welgevonden	610	Erum5510			No
ama Anaplasma marginale St. Maries	619	AM844			No
pub Candidatus Pelagibacter ubique	619	SAR11_0731			No
ots Orientia tsutsugamushi Boryong	675	OTBS_1557			No
rpr Rickettsia prowazekii Madrid E	683	RP256			No
eaa Endosymbiont of Acanthamoeba sp. UWC8	698	I862_02560			No
rbt Rickettsiales bacterium Ac37b	720	NOVO_02255			No
las Candidatus Liberibacter asiaticus psy62	723	CLIBASIA_03190			No (RWW)
magq Magnetospira sp. QH-2	804	MGMAQ_2469			Yes
pgv Polymorphum gilvum	823	SL003B_2049			Yes
apb Candidatus Puniceispirillum marinum	846	SAR116_1034			Yes
pla Parvibaculum lavamentivorans	863	Plav_3278			Yes
phl Pelagibacterium halotolerans	864	KKY_1548			Yes
thal Candidatus Endolissoclinum faulkneri	864	A1OE_466			Yes
mes Chelativorans sp. BNC1	868	Meso_1711			Yes
pde Paracoccus denitrificans	869	Pden_2965			Yes
bhe Bartonella henselae Houston-1	880	BH08720			Yes
swi Sphingomonas wittichii	880	Swit_3848			Yes
sal Sphingopyxis alaskensis	881	Sala_3172			Yes
cna Croceicoccus naphthovorans	884	AB433_07985			Yes
rru Rhodospirillum rubrum ATCC 11170	889	Rru_A2149			Yes
jan Jannaschia sp. CCS1	890	Jann_2539			Yes
mmr Maricaulis maris	890	Mmar10_1296			Yes
bme Brucella melitensis bv. 1 16M	891	BMEI1057			Yes
sjp Sphingobium japonicum	892	SJA_C1-07660			Yes
gox Gluconobacter oxydans 621H	892	GOX1731			No
aex Asticcacaulis excentricus	894	Astex_2996	336	Astex_1195	Yes (894), No (336)
pzu Phenylobacterium zucineum	896	PHZ_c1842			Yes
deq Devosia sp. H5989	898	XM25_08200			Yes
ccr Caulobacter crescentus CB15	898	CC_1877			Yes
hba Hirschia baltica	904	Hbal_1895			Yes
acr Acidiphilium cryptum	906	Acry_0388			Yes
hne Hyphomonas neptunium	909	HNE_0673			Yes
bsb Brevundimonas subvibrioides	910	Bresu_2193	342	Bresu_1711	Yes (910), No (342)
eli Erythrobacter litoralis	910	ELI_06775			Yes
zmo Zymomonas mobilis subsp. mobilis ZM4	912	ZMO0193			Yes
aay Altererythrobacter atlanticus	913	WYH_00645			Yes
sno Starkeya novella	915	Snov_1332			Yes
rde Roseobacter denitrificans	919	RD1_2267			Yes
sme Sinorhizobium meliloti 1021	924	SMc01336			Yes
oan Ochrobactrum anthropi ATCC 49188	926	Oant_2268			Yes
ptp Planktomarina temperata	927	RCA23_c13330			Yes
ead Ensifer adhaerens	932	OV14_2379			Yes
oat Octadecabacter antarcticus	933	OAN307_c33460			Yes
nar Novosphingobium aromaticivorans	935	Saro_2318			Yes
rsp Rhodobacter sphaeroides 2.4.1	936	RSP_2131	347	RSP_0624	No
kvu Ketogulonicigenium vulgare Y25	941	EIO_1096			Yes
gbe Granulibacter bethesdensis CGDNIH1	949	GbCGDNIH1_0283	381	GbCGDNIH1_1028	Yes (949), No (381)
rpm Rhodospirillum photometricum	950	RSPPHO_01687			No
mcg Methyloceanibacter caenitepidi	960	GL4_1742	170	GL4_3267	Yes (960), No (170)
ret Rhizobium etli CFN 42	961	RHE_CH01645			Yes
mey Martelella endophytica	972	TM49_13015			Yes
hoe Hoeflea sp. IMCC20628	973	IMCC20628_02279			Yes
atu Agrobacterium fabrum	977	Atu1339			Yes
mai Micavibrio aeruginosavorus ARL-13	977	MICA_356			Yes
pga Phaeobacter inhibens	978	PGA1_c13450	340	PGA1_c31110	Yes (978), No (340)
gxy Gluconacetobacter medellinensis NBRC 3288	978	GLX_24260			Yes
mlo Mesorhizobium loti	984	mll0212			Yes
mgm Magnetococcus marinus	984	Mmc1_1776			No
lmd Leisingera methylohalidivorans	992	METH_10485			Yes
ngl Neorhizobium galegae bv. officinalis bv. offici	993	RG1141_CH12430			Yes
sil Ruegeria pomeroyi	993	SPO1893			Yes
msc Methylocystis sp. SC2	994	BN69_0782			Yes
apt Acetobacter pasteurianus IFO 3283-01	997	APA01_19870			Yes
cid Celeribacter indicus	1000	P73_1743			Yes
txi Thalassospira xiamenensis	1001	TH3_11950	477	TH3_15200	Yes (1001), No (477)
bid Beijerinckia indica	1005	Bind_0931			Yes
malg Marinovum algicola	1010	MALG_01460	339	MALG_00637	Yes (1010), No (339)
psf Pseudovibrio sp. FO-BEG1	1019	PSE_3473			Yes
hdn Hyphomicrobium denitrificans ATCC 51888	1025	Hden_0842			Yes
dsh Dinoroseobacter shibae	1026	Dshi_2340			Yes
bja Bradyrhizobium diazoefficiens USDA 110	1032	bll4305			Yes
gdi Gluconacetobacter diazotrophicus PAl 5	1036	GDI_2343			Yes
pbr Parvularcula bermudensis	1038	PB2503_09794			Yes
fil Filomicrobium sp. W	1047	BN1229_v1_0024			Yes
azl Azospirillum sp. B510	1047	AZL_005310			Yes
mex Methylobacterium extorquens PA1	1049	Mext_3659			Yes
rva Rhodomicrobium vannielii	1049	Rvan_3063			Yes
rpa Rhodopseudomonas palustris CGA009	1051	RPA2450			Yes
nwi Nitrobacter winogradskyi	1059	Nwi_1720			Yes
xau Xanthobacter autotrophicus	1080	Xaut_4668			Yes
msl Methylocella silvestris	1080	Msil_0729			Yes
azc Azorhizobium caulinodans	1093	AZC_3627			Yes
tmo Tistrella mobilis	1120	TMO_c0704			Yes
oca Oligotropha carboxidovorans OM5	1123	OCAR_5985			Yes
paca Candidatus Paracaedibacter acanthamoebae	Not found				
caq Candidatus Caedibacter acanthamoebae	Not found				
hci Candidatus Hodgkinia cicadicola Dsem	Not found				
